# Efficient Warming Textile Enhanced by a High‐Entropy Spectrally Selective Nanofilm with High Solar Absorption

**DOI:** 10.1002/advs.202204817

**Published:** 2022-11-29

**Authors:** Cheng‐Yu He, Peng Zhao, Hong Zhang, Kai Chen, Bao‐Hua Liu, Zhong‐Wei Lu, Yang Li, Pei‐Qing La, Gang Liu, Xiang‐Hu Gao

**Affiliations:** ^1^ Laboratory of Clean Energy Chemistry and Materials, State Key Laboratory of Solid Lubrication Lanzhou Institute of Chemical Physics Chinese Academy of Sciences Lanzhou 730000 China; ^2^ State Key Laboratory of Advanced Processing and Recycling of Nonferrous Metals School of Materials Science and Engineering Lanzhou University of Technology Lanzhou 730050 China; ^3^ Center of Materials Science and Optoelectronics Engineering University of Chinese Academy of Sciences Beijing 100049 China; ^4^ State Key Laboratory of Fluid Power and Mechatronic Systems School of Mechanical Engineering Zhejiang University Hangzhou 310027 China

**Keywords:** high‐entropy, personal thermal management, smart textile, solar warming, spectrally selective film

## Abstract

Solar and radiative warming are smart approaches to maintaining the human body at a metabolically comfortable temperature in both indoor and outdoor scenarios. Nevertheless, existing warming textiles are ineffective in frigid climates because the solar absorption of selective absorbing coating is significantly reduced when coated on rough textile surface. Herein, for the first time, high‐entropy nitrides based spectrally selective film (SSF) is introduced on common cotton through a one‐step magnetron sputtering method. The well‐designed refractive index gradient enables destructive interference effects, offering a roughness‐insensitive high solar absorptance (92.8%) and low thermal emittance (39.2%). Impressively, the solar absorptance is 9.1% higher than the reported best‐performing selective nanofilm‐based textile. As a result, such a textile achieves a record‐high photothermal conversion efficiency (82.2% under 0.6 suns, at 0 °C). This textile yields a 3.5 °C drop in the set‐point of indoor air‐conditioner temperature. Besides, in a winter morning with an air temperature of 7.5 °C, it warms up the human skin by as large as 12 °C under weak sunlight (350 W m^−2^). More importantly, such a superior radiative warming performance is achieved by engineering the widely used cotton without compromising its breathability and durability, showing great potential for practical applications.

## Graphical abstract

High‐entropy engineering is adapted to the design of high‐efficiency solar warming textile. The high‐entropy coating offers both roughness‐insensitive high solar absorption and low IR emissivity even coated on cotton. The coating is prepared by a facile one‐step sputtering method, yielding outstanding adhesion and anti‐oxidation properties. All these merits make it suitable for solar and radiative warming without compromising its wearability.

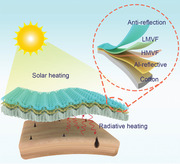

## 1 Introduction

Warming up the human body at a metabolically comfortable temperature is pivotal for maintaining the basic physiological function of human beings, as well as the prevention of cold‐related injuries and illnesses. Nevertheless, indoor space heating accounts for a considerable portion of global energy consumption.^[^ ^1^, ^2^ ^]^ Current space heating strategies such as air conditioners heat the large empty space of the entire building, wasting substantial electricity and hindering the transition toward a low carbon world.^[^ ^3^ ^]^ Moreover, these space heating strategies are ineffective for outdoor heating because of the variable location and enormous outdoor space. Outdoor activities are indispensable in our daily life, including outdoor working, sports, travelling, and so on, although harsh climates will bring about health risks. Wearing heavy clothes can help maintain our body temperature and circumvent discomfort and hypothermia, but they are somewhat inconvenient and may reduce work productivity.

**Figure 1:**
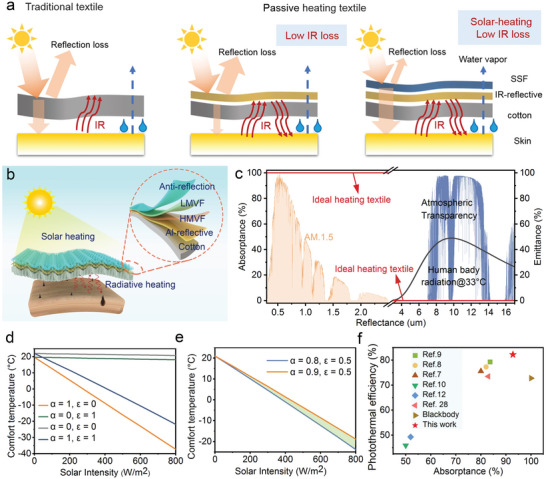
Working mechanism and advantage of ZrNbMo‐Al‐N based SSF. a) A comparison of heat inflow and outflow resulting from radiation and solar irradiance for traditional textile, IR‐reflective textiles, and solar heating textile. b) Schematic illustration of a reactive magnetron cosputtering system used in this work. c) The absorptance and emittance spectra of the ideal radiative warming textile. AM1.5 (normalized AM1.5 solar spectrum), atmospheric transparency window, and human body radiation at 33 °C. d) Calculated comfort temperatures for those textiles with different optical properties along with different solar radiation intensities. e) Comfort temperature comparison for the textiles with the same emittance but different solar absorptance under different solar radiation intensity. f) Solar absorptance and photothermal efficiency compared with previously reported textiles with emittance less than 50%.^[^ ^7^, ^8^, ^9^, ^10^, ^12^, ^28^ ^]^

Recently, personal heat management as a burgeoning field has aroused enormous attention, providing a smart thermal regulation strategy rather than heating or cooling the whole building.^[^ ^4^, ^5^, ^6^, ^7^, ^8^, ^9^, ^10^, ^11^, ^12^, ^13^, ^14^, ^15^, ^16^, ^17^, ^18^, ^19^, ^20^ ^]^ For a stationary human body, infrared radiation (IR) leads to about 50% of heat loss.^[^ ^21^ ^]^ Unfortunately, traditional textile materials such as cotton and polyester have high IR emissivity (*ε*_cloth_ = 90%).^[^ ^9^ ^]^ Although increasing the textile thickness can decrease the heat loss through convection and conduction, massive radiative heat loss is unavoidable (**Figure** **1**a). Reducing the IR emissivity of cloth surfaces is an effective strategy to realize passive radiative personal warming. For instance, the commercially available Mylar blanket uses a dense metallic film to reduce the IR thermal loss, but its poor breathability limits daily wearing.^[^ ^8^ ^]^ Several attempts have been made to develop low‐emissivity textiles with improved breathability, which potentially reduce indoor environment set‐points and save building heating energy.^[^ ^4^, ^5^, ^6^ ^]^

In a typical outdoor situation, however, both traditional and low‐emissivity textiles cannot meet the huge heating needs. The presence of an atmospheric window (8–13 µm), which corresponds to the peak wavelength (9.7 µm) of human body radiation at 33 °C (Figure 1c), allows the human body dissipates heat to the cold outer space (*T* = 2.7 K), leading to enhanced radiative loss.^[^ ^22^ ^]^ Moreover, the increased convective heat coefficient in the outdoor environment poses another challenge to maintain our thermal comfort.^[^ ^18^ ^]^ Fortunately, if outdoor solar irradiation can be efficiently harvested by textiles, it is possible to realize effective personal warming in a green manner. Although great efforts were made in modifying textile surfaces to increase solar heating capability, most solar harvesting performance evaluations are conducted at air temperatures above 20 °C.^[^ ^7^, ^8^, ^10^ ^]^ Even worse, the relatively low solar radiation intensity in winter poses an additional challenge to these textiles in generating efficient solar heating effects. Moreover, most previous radiative warming textiles are made from expensive conductive fabrics or nanoporous polyethylene (Nano‐PE) textiles.^[^ ^8^, ^9^ ^]^ As a result, large‐scale applications are currently not feasible. Furthermore, there is always a dilemma between personal thermal management ability and wearability, which should also be considered during the fabrication process. In short, a highly efficient solar/radiative warming strategy based on widely used textile materials with low cost and great wearability is highly desired.

Spectrally selective films (SSFs), which are capable of effectively absorbing solar energy in the solar spectrum (0.3–2.5 µm) and suppressing IR thermal loss in the IR region (2.5–17 µm) simultaneously, show great promise to overcome those outdoor personal heating limitations.^[^ ^23^, ^24^, ^25^, ^26^, ^27^ ^]^ Great attempts have been made to adopt SSFs to the design of solar‐warming textiles. However, due to the larger surface roughness of cottons than flat metals, almost all SSFs lose their high efficiency when coated on cotton or other textile materials. The high solar absorption of these SSFs was enabled by the strong resonance coupling between the absorbing nanoparticles/films and the bottom flat metal film, which is highly sensitive to the roughness of the bottom metal. For instance, Li et al. and Zhang et al. demonstrated that the solar absorptances of their plasmonic metamaterials are notably reduced when coated on nylon 66 porous films (from 95% to 45%) and cottons (from 95% to 83.7%) compared to flat metal substrates.^[^ ^9^, ^23^ ^]^ Alternatively, Wang et al. demonstrated that the solar absorptance of their MoO*_x_*‐based multilayer SSFs show a notable decrease from 93% to 83% when coated on cotton compared to flat metal films.^[^ ^28^, ^29^ ^]^ To address this commonplace and critical issue, for the first time, we resorted to high‐entropy strategies in the development of SSFs for textiles. High‐entropy nitride is a category of up‐and‐coming photothermal materials due to its unique and fascinating structural characteristics, and large compositional space.^[^ ^30^, ^31^, ^32^ ^]^ It remains, however, a significant challenge to achieve desired spectral selectivity on rough and porous substrates.

In this work, we fabricated a multilayer high‐entropy nitride based SSF on a cotton through a one‐step magnetron sputtering method. The resultant textile embraces a superior spectral selectivity (*α*/*ε* = 92.8%/39.2%), which simultaneously enables solar heating and passive radiative warming. Finite‐difference time‐domain (FDTD) simulation reveals that the outstanding sunlight harvesting ability benefits from the collective effects of destructive interference and intrinsic absorption of high‐entropy nitrides. Significantly, compared to pristine cotton, such a textile allows a 3.5 °C decrease in the set‐point of indoor air‐conditioner temperature, leading to significantly reduced energy consumption. Furthermore, when covered on human skin, this textile enables a real skin temperature rise of around 12 °C at 350 W m^−2^ of solar radiation under air temperatures of about 7.5 °C. More importantly, this outstanding warming ability of the textile does not compromise the breathability and durability of cotton. Overall, the outstanding radiative warming performance, low cost, and great wearability make the SSF‐decorated textile promising for practical applications in personal thermal management, especially in frigid climates.

## 2 Results and Discussion

### 2.1 Theoretical Foundation for Radiative Warming

In outdoor scenarios, the IR transparent atmospheric window (8–13 µm) imposes more burden on radiative warming textiles than the indoor cases. The single passive radiative heating textile cannot efficiently reduce human body comfort temperature. Smaller comfort temperature implies that the textile could endure lower outdoor temperature without compromising human thermal comfort; in other words, the textile possesses better radiative warming performance. Harvesting solar energy through the textile surface is a promising approach to decreasing the comfort temperature. Increasing solar absorptance and suppressing thermal emittance are two efficient approaches to improving solar thermal efficiency. Under solar radiation, an ideal radiative warming textile should have unity absorption in the solar spectrum and zero emission in the mid‐IR region (Figure 1c).

To highlight the importance of optical properties for radiative warming textile, we thoroughly investigated the influences of absorptance and emittance on comfort temperatures. The comfort temperatures in a variety of scenarios can be determined by a steady‐state heat transfer model (see the Supporting Information).^[^ ^9^, ^17^ ^]^ The results demonstrate that the utilization of solar radiation can enable a significant reduction of comfort temperatures (Figure 1d). For the ideal radiative warming textile, even a small amount of solar irradiance can make human bodies feel comfortable in frigid weather. For instance, at an ambient temperature of less than 0 °C, a human wearing the textile can realize a comport temperature under 300 W m^−2^ solar illumination. The textile with *α* = 1 and *ε* = 1, in which the passive radiative warming mechanism is eliminated, still maintains outstanding solar warming ability. Under weak sunlight (300 W m^−2^) illumination, it enables the human body maintaining the comfort temperature when the air temperature is around 9 °C. Moreover, we also performed the theoretical analysis to highlight the importance of solar absorptance for solar radiative warming. Figure 1e shows the comfort temperature variation of two textiles with the same IR emittance (*ε* = 0.5) but different solar absorptances. For example, by increasing solar absorptance from 0.8 to 0.9, the human body still feels comfortable although the ambient temperature is decreased for 2.1 °C with solar irradiance of 300 W m^−2^, suggesting that solar absorptance plays a crucial role in keeping the human body comfortable in outdoor ambient.

Therefore, based on the above‐mentioned theoretical analysis, we proposed an efficient radiative warming textile by adopting a trilayer high‐entropy nitride based SSF to Al‐decorated cotton, schematically illustrated in Figure 1b. The SSF consists of a double‐layer ZrNbMo‐Al‐N with gradient optical properties that can efficiently harvest solar energy through destructive interference effects. The antireflection layer Si_3_N_4_ could decrease solar reflectance by offsetting the refractive index difference between the ZrNbMo‐Al‐N and air. Nevertheless, the spectral selectivity of the as‐prepared SSF relies on the dense and continuous metal substrate. It is therefore challenging to simultaneously realize high solar absorptance and low emittance on rough and porous common cotton. To this end, an Al nanofilm was deposited on the cotton to increase its IR reflectance before the deposition of the tri‐layer SSF. The resultant radiative warming cotton displays an excellent solar absorptance of 92.8%, accompanied by a low IR emittance of 39.2%. It is worth noting that the solar absorptance of the SSF‐decorated textile is 9.1% higher than that of the best‐performing radiative warming textiles (Figure 1f). The photothermal conversion efficiency is calculated to fairly compare the performance of our SSF‐decorated cotton with those reported warming textiles. In this calculation, the solar radiation intensity was set to 600 W m^−2^, while the ambient temperature was 0 °C, which are typical winter weather conditions. Due to its outstanding solar absorptance, the SSF‐decorated cotton yields an outstanding photothermal conversion efficiency (82.2%), surpassing state‐of‐the‐art solar radiative warming textiles.^[^ ^7^, ^8^, ^9^, ^10^, ^12^, ^28^ ^]^ In a comparison, the blackbody shows a lower efficiency (72.8%) due to its quite high emittance that results in massive thermal radiation heat loss. This highlights the importance of this work to meet solar radiative warming requirements in freezing weather.

### 2.2 Optimization and Preparation on a Rigid Substrate

**Figure 2:**
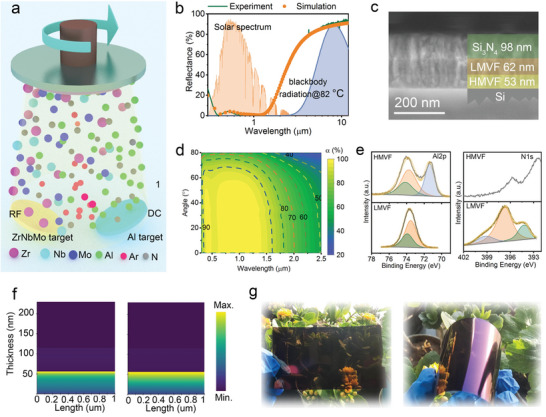
Fabrication and flexible application of the ZrNbMo‐Al‐N based SSF. a) Schematic illustration of a reactive magnetron cosputtering system used in this work. b) Experimental and fitted reflectance profiles of the trilayer structured SSF and the solar intensity and blackbody radiation at 82 °C. c) Cross‐section SEM image of the as‐deposited SSF on a Si substrate. d) Calculated solar absorptance versus wavelengths and incidence angles. e) High‐resolution XPS spectra of Al 2p and N 1s of the HMVF and LMVF layers. f) The absorbed power distribution of the SSF at the wavelengths of 0.38 and 1.3 µm, which corresponds to two reflectance dips in the reflectance spectrum. g) The optical photographs of the SSF deposited on the PET sheet show the bendable and scalable nature.

The SSF was first optimized on a rigid stainless steel (SS) substrate. As displayed in **Figure** **2**a, ZrNbMo‐Al‐N films are prepared by a combinatorial magnetron cosputtering approach that is easy to scale up. The optical properties of ZrNbMo‐Al‐N film can be tuned by changing the N_2_ flow rate. According to the previous report,^[^ ^31^ ^]^ a high N_2_ flow rate leads to increased dielectric properties of the film. Accordingly, we devised an optical gradient triple‐layer SSF, comprising a high metal volume fraction (HMVF) ZrNbMo‐Al‐N layer as the main absorption layer, a low metal volume fraction (LMVF) ZrNbMo‐Al‐N layer that facilitates destructive interference, and a dielectric Si_3_N_4_ as an antireflection layer. During the deposition process, the optimization of deposition parameters for HMVF and LMVF layers is very crucial. Therefore, we deposited a series of ZrNbMo‐Al‐N films on glass substrates with varying N_2_ flow rates and then measured the reflectance (*R*) and transmittance (*T*) spectra (Figure S1, Supporting Information). Subsequently, by fitting the *R* and *T* spectra, the optical constants (refractive index *n* and extinction coefficient *k*) could be derived. As the N_2_ flow rate is less than 3 sccm, both *n* and *k* increase with wavelengths, suggesting the metallic feature. Further increasing N_2_ flow rate significantly reduces the *n* and *k*, endowing those films with dielectric properties.

Based on the above calculated optical constants, we optimized the reflectance spectrum of trilayer SSF on SS substrate using the commercial CODE software. The optical constants of the SS substrate and Si_3_N_4_ film were referred to our previous work.^[^ ^30^ ^]^ According to the optimized parameters such as the N_2_ flow rate and layer thickness, a triple‐layer SSF was successfully fabricated, which shows a high solar absorptance of 96.2% and suppressed thermal emittance of 8.8%. Each layer collaboratively contributes to enhanced solar absorption (Figure S2, Supporting Information). In addition, the experimental profile agrees well with the simulated one, validating the excellent reproducibility of the fabrication process (Figure 2b). The cross‐section scanning electron microscope (SEM) morphology of the as‐deposited SSF confirms the three‐layer structure with evident interfaces (Figure 2c). The thicknesses of HMVF, LMVF, and Si_3_N_4_ layers are measured to be 53, 62, and 98 nm, respectively. Each element is uniformly distributed according to elemental mappings for HMVF and LMVF layers (Figures S3 and S4, Supporting Information). SEM and 3D atomic force microscope (AFM) morphologies of the as‐deposited SSF demonstrate the flattened and compact surface (Figures S5 and S6, Supporting Information). Moreover, the SSF enables omnidirectional solar absorption with incidence angles of 0–60°, allowing efficient solar energy harvesting regardless of the time of day (Figure 2d and Figure S7, Supporting Information). Also, we performed an FDTD simulation to discover the underlying solar harvesting mechanism behind the multilayer nanofilm structure (Figure 2f). Specifically, the representative wavelengths are selected to be 0.38 and 1.3 µm, respectively, which correspond to two reflectance dips in the reflectance spectrum. The absorbed power is mainly confined at the interface between HMVF and LMVF layers, demonstrating that destructive interference due to the gradient optical constants is the main reason for the intense absorption. For the incident light at 1.3 µm, the majority of photons were dissipated by the high‐entropy nitrides interface and the HMVF layer due to the increased penetration depth. For high‐entropy materials composed of transition metals, the d electron intensities around the Fermi level are enhanced compared with the low‐entropy counterparts;^[^ ^33^ ^]^ besides, high‐entropy nitrides composed of transition metals have narrow bandgaps.^[^ ^34^, ^35^ ^]^ Therefore, d‐d transitions are easily activated, that is, the d‐band electrons with energy above the bandgap transit from the valence band to the conduction band, thereby enhancing solar harvesting capability in the UV–vis–NIR region.^[^ ^36^ ^]^ Such d‐d band transition induced solar absorption is intrinsic and roughness‐insensitive. Moreover, lattice vibration due to the lattice defect of HENs fosters phonon scattering, further increasing solar absorption.^[^ ^37^ ^]^ Consequently, the collective effects of destructive interference and intrinsic absorption of high‐entropy nitride ZrNbMo‐Al‐N boost sunlight harvesting ability.

To reveal the bonding states of metallic elements, X‐ray photoelectron spectroscopy (XPS) is carried out for the high‐entropy nitride ZrNbMo‐Al‐N. Considering that the passive film was formed on the surface (Figure S8, Supporting Information), we performed 20 nm Ar^+^ etching for those as‐deposited films to remove surface impurities. For the Al 2p spectrum (Figure 2e), the peaks at 71.4, 73.7, and 74.1 eV are associated with Al^0^, Al nitridation, and Al oxidation states, respectively;^[^ ^38^, ^39^ ^]^ in addition, no metallic Al is observed for LMVF. N 1s spectrum verifies low nitrogen content in HMVF; while it could be deconvoluted into three peaks in LMVF, corresponding to N—O (398.3 eV), metal nitrides (396 eV), and N—N (393.7 eV), respectively.^[^ ^40^ ^]^ High‐resolution XPS spectra of Nb 3d, Zr 3d, and Mo 3d can be seen in Figure S9 (Supporting Information). The results demonstrate the oxidized nature of ZrNbMo‐Al‐N and reveal that the strong metallic properties of HMVF result from the existence metal state; moreover, increasing nitrogen content contributes to higher binding energies of metal nitridation and oxidation states.

To probe the application potential on a flexible substrate, the ZrNbMo‐Al‐N based SSF was deposited on polyethylene terephthalate (PET) sheet with optimized parameters above. As‐deposited PET/SSF shows good solar absorptance in the solar spectrum range (Figure S10, Supporting Information). Figure 2g depicts the optical photographs, suggesting that the proposed SSF could enable large‐area fabrication on a flexible substrate. Benefitting from its thin thickness (213 nm) and advanced magnetron sputtering technology, the SSF integrates well with the PET sheet, which could withstand immense bending, further expanding its practical applications. However, low IR reflection aggravates heat loss through IR emission. Accordingly, to strengthen solar radiative warming on cotton by the SSF, it is of significant importance to introduce an IR‐reflective layer.

### 2.3 Passive Radiative Warming Performance

**Figure 3:**
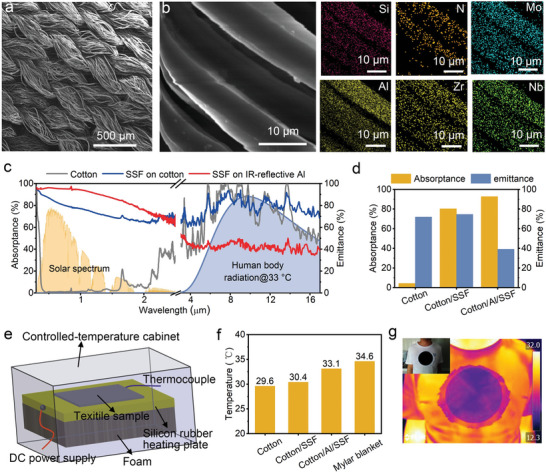
Microstructure, optical properties, and indoor application of the SSF‐decorated cotton. a) Surface SEM topography of the SSF‐decorated cotton, b) corresponding amplifying region, and elemental mappings of Si, N, Mo, Al, Zr, and Nb. c) Reflectance spectra of cotton, SSF/cotton, SSF/Al/cotton, and d) the corresponding solar absorptance and thermal emittance. e) Schematic of a home‐made experimental setup for indoor passive radiative heating performance tests, the temperature is 20 °C. f) Final temperatures of skin simulator and the ones covered with cotton, cotton/Al/SSF, and Mylar blanket. g) Visible passive radiative heating evaluation of a human body covered with SSF/Al/cotton in indoor ambient.

On the basis of the above consideration, we choose easy‐to‐get common cotton as substrate (its surface morphologies can be seen in Figure S11, Supporting Information), the Al film as an IR reflection layer deposited on the cotton, and the above‐optimized trilayer SSF boosting solar energy harvesting. By changing deposition time, the thickness of the Al film can be easily controlled and then generates different optical properties (Figure S12, Supporting Information). As the deposition time is on a par with 24 min, corresponding to 210 nm thickness (Figure S13, Supporting Information), the cotton/Al/SSF textile reaches an optimal spectral selectivity (*α* = 92.8%; *ε* = 39.2%). **Figure** **3**a displays the surface SEM image of the as‐deposited textile. The cellulose fibers with a diameter of around 11 um crosslink with each other and form a porous structure, making the SSF‐decorated cotton retain good breathability and durability of the original cloth. The diameter of those fibers in textile is distributed among the IR wavelengths, which promotes IR absorption via scattering and diffraction effects, therefore leading to an enhanced IR emittance. On the other hand, at the IR wavelengths regime, the porous structures form a continuous refractive index gradient between air and medium, which decreases the IR reflection effectively.^[^ ^40^ ^]^ As a result, the resulting textile has significantly increased thermal emittance compared with that on rigid SS substrate.^[^ ^41^ ^]^ Figure 3b shows an amplifying region and the corresponding elemental mappings, from which each element is homogeneously distributed. As shown in Figure 3c, the original cotton shows high reflectance in the solar spectrum while low reflectance in the IR region, which are not favorable to passive radiative heating or solar heating. When depositing the SSF on the cotton, the solar absorption is significantly elevated, but heat loss is still significant because IR light through SSF is absorbed by cotton. Sandwiching an Al film as an IR reflection layer greatly reduces heat loss through radiation. Eventually, the resulting SSF‐decorated cotton achieves a high solar absorption and a suppressed thermal emittance (Figure 3d).

In previous works, many kinds of excellent SSFs were developed and some of them were prepared by low‐cost coating technologies.^[^ ^42^ ^]^ For example, the black copper and black chromium‐based coatings already achieved 96%–98% absorptance and 2%–10% emittance at 100 °C using scalable electrodeposition methods.^[^ ^43^, ^44^ ^]^ However, those outstanding optical properties are all achieved on flat substrates such as polished stainless steel or metal substrates. Otherwise, those materials will lose spectral selectivity due to the breaking of resonance conditions. It is challenging to deposit those selective coatings on flexible porous common textiles by low‐cost techniques such as electrodeposition or spray coating, which may result in poor adhesion and uniformity. Moreover, achieving such excellent spectral selectivity needs to rigorously control the thicknesses of each layer, which poses another significant challenge. In this context, the one‐step magnetron sputtering method will avoid those drawbacks. The thin film obtained by sputtering exhibits numerous strengths, such as high purity, density, uniformity, and repeatability. Moreover, scalable fabrication of sputtering technology has been achieved by industrial magnetron sputtering through a roll‐to‐roll technology.^[^ ^45^ ^]^

To understand the advantage of SSF‐decorated cotton in terms of its low IR emissivity characteristic, we build a simple device to examine the indoor passive radiative heating performance (Figure 3e). The cotton and commercially available Mylar blanket (see optical spectra in Figure S14, Supporting Information) are used as references. Those samples are respectively placed in a temperature/humidity controllable room consisting of a polymer sheet. Artificial skin (see optical spectra in Figure S15, Supporting Information) with an emittance close to real skin was attached to a silicone heater to simulate human skin. The silicone heater can control output power by a CD power supply, providing a constant heat flux of 110 W m^−2^ for simulating human body metabolic heat. The room temperature and relative humidity, respectively, maintain at 20 °C and 46% during the experimental process. As shown in Figure 3f, the final equilibrium temperature of the skin covered by SSF‐decorated cotton reaches up to 33.1 °C, visibly higher than that of temperature covered by cotton (29.6 °C). The significantly increased heating performance of SSF‐decorated cotton results from its low thermal emissivity, hindering radiative heat loss. Although Mylar blanket capture higher temperature, it sacrifices its breathability, restricting practical use.

Moreover, to intuitively corroborate the outstanding passive radiative heating performance of cotton/Al/SSF, we covered it on the human body and captured the optical and IR thermal images (Figure 3g). Prior to imaging, the cotton is at thermal equilibrium with the human body. The optical image shows dark black color, corresponding to the superior absorptivity in the visible light region. From the thermal imaging, when covered by cotton/Al/SSF, the surface temperatures are much lower than in other areas of the body. The low surface temperature suggests that both emitted and reflected energy is extra low, which pronouncedly decreases IR heat loss, thereby warming the human body. Overall, passive radiative warming is achieved with readily available cotton and a facile one‐step magnetron sputtering, which expands its application potential, thereby lessening our dependence on fossil fuel.

### 2.4 Solar Radiative Warming Performance

**Figure 4:**
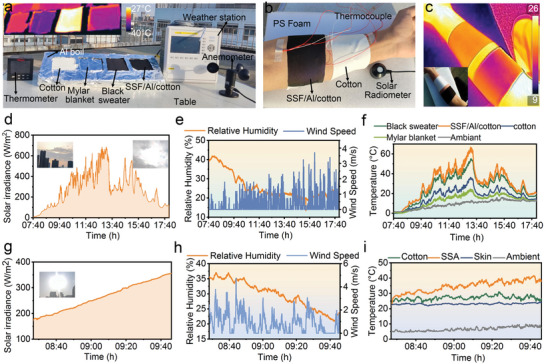
Solar heating performance of the SSF‐decorated cotton. a) Digital photograph of a home‐made solar heating apparatus placed on the rooftop (February 18, 2021, Lanzhou China,103:50 E, 36:03 N) to evaluate the solar radiative ability; the inset is the corresponding IR image. b) Optical image of solar radiative warming performance evaluation for the cotton and SSF‐decorated cotton on a real human. c) IR and optical photographs of the cotton and SSF‐decorated cotton on a real human. d) Recorded solar irradiance intensity, e) relative humidity and wind speed during the daytime experiment, and (f) corresponding teal‐time temperatures of the skin simulator covered with different textiles. g) Recorded solar irradiance intensity and h) relative humidity and wind speed during the measurement on real human skin, i) corresponding real‐time temperatures of the skin covered with different textiles.

To highlight the excellent solar warming performance of the cotton/Al/SSF textile, we first construct an apparatus for recording the surface temperatures of artificial skin covered by different textiles (**Figure** **4**a). The apparatus was placed on one roof (February 18, 2021, Lanzhou, China,103:50 E, 36:03 N) on a cloudy day, facing to sun, and exposed to wind flows. In addition to surface temperatures, the solar irradiance (Figure 4d), relative humidity and wind speed (Figure 4e), and ambient temperature are monitored during the measurement process. Figure 4f displays real‐time artificial skin temperatures covered with various textiles as well as ambient temperature. It is worth mentioning that a black sweater (see reflectance spectrum and thickness in Figures S16 and S17 (Supporting Information), with *α* = 83%, *ε* = 91%, and 2.184 mm in thickness) is used as a reference with a much larger thickness than cotton/Al/SSF (0.233 mm). When the solar radiation intensity is less than 80 W m^−2^, there is no advantage for the surface temperature of the artificial skin covered by cotton/Al/SSF textile over the black sweater counterpart. Obvious temperature differences can be seen when the solar radiation intensity is more than 100 W m^−2^. Although the cotton/Al/SSF textile is more susceptible to suffering from a gust of wind because the thin thickness increases convective heat loss, its excellent solar energy harvesting ability allows for higher surface temperatures. As the sky becomes clear at around 12:50, artificial skin temperature covered by cotton/Al/SSF is 12 °C higher than that covered by a black sweater and even 28 °C higher than that covered by pristine cotton (Figure S18, Supporting Information). At around 13:40, the sunlight is blocked by clouds, accompanying with increasing wind speed, which collaboratively reduces the skin simulator temperatures significantly. Fortunately, the temperatures covered by cotton/Al/SSF are still far beyond that covered by cotton. The strong Mid‐IR solar energy absorption and low IR thermal loss are responsible for the high temperature. Moreover, we performed an identical test on a sunny day, which achieved a higher temperature difference (Figures S19 and S20, Supporting Information). In particular, the peak temperature of the artificial skin covered by cotton/Al/SSF reaches up to 70.5 °C, which is 11.4, 35.6, and 41.5 °C higher than that of a black sweater, cotton, and Mylar blanket, respectively. Due to low solar absorptance that limits solar energy harvesting, the artificial skin covered by Mylar blanket textile shows a very low temperature. Over the daytime tests, the cotton/Al/SSF textile shows excellent solar warming performance, suggesting a high potential for practical use in outdoor scenarios.

Furthermore, to intuitively validate the solar warming performance of the cotton/Al/SSF, we covered it on human skin and monitored real‐time temperatures and other parameters mentioned above (Figure 4b). Figure 4c shows an IR image taken after reaching a heat equilibrium between thermogenesis and exothermicity. The cotton/Al/SS textile exhibits a low surface temperature, which is lower than the cotton counterpart and far less than the skin temperature. This demonstrates a low heat loss through IR radiative and plays a good role in passive radiative warming of the human body in outdoor ambient. Figure 4g,h displays real‐time monitoring of the solar irradiance, relative humidity, and wind speed on a sunny day. Significantly, although the solar irradiance is weak, the skin covered by cotton/Al/SSF captures a high surface temperature, 15.6 °C, and 17.2 °C higher than that covered by cotton and bare skin, respectively (Figure 4i and Figure S21, Supporting Information). This temperature difference results from both high solar absorptivity which harvests much solar irradiance, and low emissivity, which reduces IR thermal loss, so that effectively warming the human body. As well, we carried out an identical human body test on a cloudy day (Figures S22 and S23, Supporting Information), which also confirms the outstanding solar radiative warming performance of the cotton/Al/SSF textile.

### 2.5 Wearability Tests

**Figure 5:**
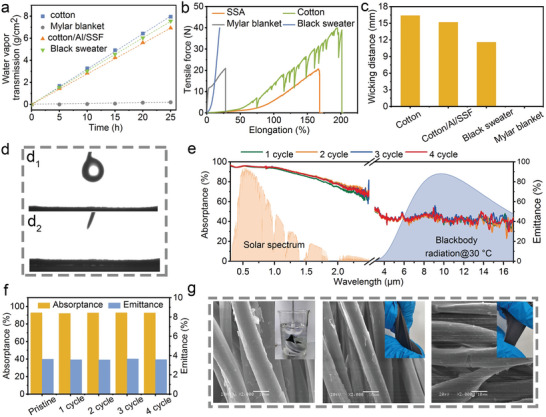
Wearability characterization. a) The water vapor transmission rate, b) tensile performance, and c) wicking distance of different textiles. d) Optical photographs of a water droplet on the cotton/Al/SSF surface, where *d*_1_ and *d*_2_ show photos before and after contact, respectively, between water droplet and textile. e) Solar absorptance and thermal emittance spectra of the cotton/Al/SSF after different washing cycles, each cycle lasted for 2 h. f) Calculated absorptance and emittance values after each cycle. g) Surface SEM morphologies after washing ten cycles, twist and stretching, respectively.

In addition to appealing radiative warming performance, the desired textile should not compromise the wearability of the original cloth. As a result, we perform a comprehensive assessment of the cotton/Al/SSF textile in terms of its breathability, mechanical strength, washability, and durability. As shown in **Figure** **5**a, the breathability tests indicate that the proposed cotton/Al/SSF textile brings forth a great water vapor transmission rate comparable to that of pristine cotton due to its porous nanowire structure. Tensile tests exhibit a maximum tolerable tensile force of 18 N, similar to that of Mylar blanket (Figure 5b).

In addition, the cotton/Al/SSF textile also shows a considerable wicking distance comparable to cotton due to the hydrophilic cellulose fibers (Figure 5c), as well as superhydrophilicity (Figure 5d), which is beneficial for perspiration evaporation and thereby improves the wearable comfort. Moreover, we also carried out the washability investigation. Those samples were placed in a beaker with distilled water and stirred with different duration cycles, each lasting 2 h. There is a slight change in the absorptance spectrum of the textile after washing one cycle, as observed in Figure 5e and Figure S24 (Supporting Information). Following two cycles of washing, the near‐IR absorption shows a slight increase. Further increasing washing durations only generates an insignificant change in those profiles. In addition, no apparent variation in the emittance spectra is observed during the overall washing cycle process. As a consequence, both solar absorptance and thermal emittance show no significant change, as shown in Figure 5f. Figure 5g and Figure S25 (Supporting Information) shows the surface SEM image that reveals a slight fracture of the nanofilm after washing for ten cycles. Fortunately, the optical performance is not attenuated. In addition, after twisting and stretching treatments, the cotton/Al/SSF textile also retains nearly intact structure, no obvious peel‐off of the coating is observed, demonstrating outstanding durability.

Significantly, the reverse side of cotton/Al/SSF textile shows reciprocal optical properties (i.e., the low solar absorptance of 39%, high thermal emittance of 80%), as shown in Figure S26 (Supporting Information). It is far‐reaching in response to sharply increased ambient temperatures, avoiding thermal discomfort caused by overheating. In addition, by changing the thickness of Al film, the solar absorptance and thermal emittance can be tailored according to practical warming requirements, further expanding application scenarios for the cotton/Al/SSF textile. Furthermore, to broaden the working scenarios (e.g., outdoors on cloudy days or at night), we deposited the well‐designed SSF on an Al‐decorated Nano‐PE.^[^ ^4^, ^8^ ^]^ Benefitting from the relatively smooth surface, the low thermal emittance of 15.6% and high absorptance of 94.1% are enabled simultaneously, as shown in Figure S27 (Supporting Information), both of which are better than those on common cotton. And its thermal emittance is comparable to those of state‐of‐the‐art warming textiles, making our warming textile more suitable for warming the human body when sunlight is unavailable.

## 3 Conclusions

In summary, we computationally and experimentally developed a novel trilayer high‐entropy nitride (ZrNbMo‐Al‐N) based SSF, and deposited it on an Al‐decorated cotton with superior spectral selectivity (*α* = 92.8%; *ε* = 39.2%), making it suitable for both passive radiative warming and solar radiative warming. Impressively, compared with pristine cotton, the cotton/Al/SSF textile realizes a 3.5 °C drop in the indoor set‐point, which is of substantial significance for indoor building heating energy savings. More significantly, owing to its outstanding solar energy harvesting ability, the cotton/Al/SSF could heat the human body even at low solar irradiance intensity in severely cold weather, which was experimentally demonstrated on both artificial skin and human skin. In addition, such a good personal thermal management performance does not compromise its wearable comfort, together with the widely used cotton and the facile fabrication method, making it a fascinating candidate for ameliorating the health conditions, especially in frigid climates. Moreover, the proposed flexible high‐entropy nitride‐based SSF provides a new possibility for diverse emerging technologies where spatial thermal regulations and continuous green energy solutions are required.

## Conflict of Interest

The authors declare no conflict of interest.

## Supporting information

Supporting Information

## Data Availability

The data that support the findings of this study are available from the corresponding author upon reasonable request.
