# Supplementary material for: Efficient Warming Textile Enhanced by a High‐Entropy Spectrally Selective Nanofilm with High Solar Absorption
Source: Adv Sci (Weinh). 2022 Nov 29;10(3):2204817. doi: 10.1002/advs.202204817 (PMC9875644; doi:10.1002/advs.202204817)
Supplement: Supplementary file 1 — Supporting Information [file ADVS-10-2204817-s001.pdf]

Supporting Information

Efficient warming textile enhanced by a high-entropy spectrally selective nanofilm with high solar absorption

Cheng-Yu He, Peng Zhao, Hong Zhang, Kai Chen, Bao-Hua Liu, Zhong-Wei Lu, Yang Li, Pei-Qing La*, Gang Liu, Xiang-Hu Gao**

Dr. Cheng-Yu He, Peng Zhao, Dr. Bao-Hua Liu, Zhong-Wei Lu, Prof. Gang Liu, Prof. Xiang-Hu Gao
Laboratory of Clean Energy Chemistry and Materials, State Key Laboratory of Solid Lubrication, Lanzhou Institute of Chemical Physics, Chinese Academy of Sciences, Lanzhou 730000, China
E-mail: gaoxh@licp.cas.cn
Prof. Pei-Qing La
State Key Laboratory of Advanced Processing and Recycling of Nonferrous Metals, School of Materials Science & Engineering, Lanzhou University of Technology, Lanzhou 730050, China
E-mail: pqla@lut.cn
Dr. Hong Zhang, Dr. Kai Chen
Center of Materials Science and Optoelectronics Engineering, University of Chinese Academy of Sciences, Beijing 100049, China
Dr. Yang Li
State Key Laboratory of Fluid Power and Mechatronic Systems, School of Mechanical Engineering, Zhejiang University, Hangzhou, 310027, China
E-mail: ylidn@connect.ust.hk

EXPERIMENTAL METHODS

Fabrication: To optimize the optical performance, single-layer ZrNbMo-Al-N films are deposited on glass with different N_2 flow rates by a co-sputtering system (Kurt J. Lesker, USA). Based on the computationally optimized parameters, ZrNbMo-Al-N and Si_3N_4 films are successively deposited on stainless steel (SS) substrate. This is achieved by a nominally equimolar ZrNbMo (purity 99.9%), Al (purity 99.9%), and Si (purity 99.9%) target under $Ar/N_2/O_2$ atmosphere. ZrNbMo and Si_3N_4 are powered by radio-frequency (RF) power, while the Al target is powered by direct current (DC) power. During the magnetron sputtering

process, the target current and substrate bias keep constant, and substrate temperature does not change at room temperature. Before being placed into the vacuum chamber, all SS substrates were cleaned with alcohol, acetone, and de-ionized water in an ultrasonic agitator. The base pressure was pumped down to 5.5 × 10^{-6} mtorr by a cryopump. The thickness of those films was controlled by the method that calculates the film growth rate through deposition time. The detailed deposition parameters can be seen in Table S1. The reflective Al film was deposited on common cotton to decrease IR thermal loss. Subsequently, the optimized tri-layer ZrNbMo-Al-N based absorber was deposited on the reflective Al decorated cotton.

Characterization: The reflectance and transmittance spectra in the solar spectrum range (0.3-2.5 μm) were measured by a Perkin Elmer Lambda 950 UV/Vis/NIR Spectrometer with an integration sphere (module 150 mm). Reflectance spectra in the infrared region (2.5-17 μm) were measured on a Bruker TENSOR 27 FT-IR Spectrometer, equipped with an integrating sphere (A562-G/Q) using a gold plate as a standard for diffuse reflectance. According to experimental spectra, the normal α_S and ε_T values were obtained by Eqs. (S1) and (S2).

$$\alpha_S = \frac{\int_{0.3\mu m}^{2.5\mu m}\left[1-R(\lambda)\right]I_{sol}(\lambda)d\lambda}{\int_{0.3\mu m}^{2.5\mu m}I_{sol}(\lambda)d\lambda} \quad \text{(S1)}$$

where λ is the specific wavelength, $R(\lambda)$ presents reflectance, and $I_{sol}(\lambda)$ is the direct normal solar irradiance which is defined according to ISO standard 9845-1, normal radiance, AM 1.5. Normal thermal emittance ε_T is equally a weighted fraction, but between emitted radiation and the Planck black body distribution, $I_b(\lambda, T)$, at temperature T.

$$\varepsilon_{\mathrm{T}} = \frac{\int_{0.3\mu m}^{17\mu m} \left[1 - R(\lambda)\right] I_b(\lambda, T) d\lambda}{\int_{0.3\mu m}^{17\mu m} I_b(\lambda, T) d\lambda} \tag{S2}$$

It is worth noting that the absorbers' reflectance spectra before and after annealing were measured at 82 °C. Accordingly, solar absorptance and thermal emittance of the absorbers at high temperatures were calculated based on the reflectance spectra.

To evaluate the solar-thermal conversion performance of cotton/Al/SSF textile in practical applications, photothermal conversion efficiency (η) was employed to quantitatively weigh the influence caused by solar absorptance (α) and high-temperature thermal emittance (ε), which is shown below:

$$\eta = \alpha - \frac{\varepsilon\sigma(\mathrm{T}^4 - T_0^{\ 4})}{CI} \tag{S3}$$

In formula (S3), C, I, and σ represent the solar concentration ratio, solar flux intensity (AM1.5G), and Stefan-Boltzmann constant. T represents the operating temperature, i.e., skin temperature, while T_0 represents ambient temperature.

Based on the measured spectra (reflectance and transmittance) of ZrNbMo-Al-N film deposited on glass, CODE software is utilized to calculate the optical constants (n, k) by fitting the experimental transmittance and reflectance spectra. X-ray diffraction (XRD) patterns were recorded on a Rigaku D/max 2400/PC diffractometer (Rigaku Corporation, Tokyo, Japan) with Cu Ka radiation (l51.5406 Å). The surface morphologies were observed by a high-resolution scanning electron microscope (SU8200, Tokyo, Japan). An atomic force microscope (AFM, Agilent 5100) is used to further study surface morphologies during the annealing process, with an area of 2×2µm2. The NanoScope Analysis 1.8 software is used to

investigate the change of the root mean square (RMS) of the annealed coatings. The Raman spectra were obtained using a Raman technique (LabRAM HR Evolution, HORIBA).

Optical models: In the case of doped semiconductors, the charge carriers set free by the donors or acceptors can be accelerated by very little energy and hence do respond to applied electric fields with frequencies in the infrared region, which the Drude model gives.[1]

$$\varepsilon_{HMVF} = \varepsilon_{back\ ground} + \varepsilon_{Drude} + \Sigma\varepsilon_{Kim} + \varepsilon_{OJL} \qquad (S4)$$

$$\varepsilon_{LMVF} = \varepsilon_{back\ ground} + \Sigma\varepsilon_{Kim} + \varepsilon_{OJL} \qquad (S5)$$

Where, the OJL model is used to show interband transitions put forward by the OLeary model where expressions for the joint density of states are given for optical transition from the valence band to the conduction band.[2,3] The Kim model is an extension of the simple harmonic oscillator for vibrational modes, which allows a continuous shift of the line shape between a Gaussian and a Lorentzian profile.[4]

FDTD simulation. A commercial finite-difference time-domain (FDTD) software package Lumerical Solution is employed to simulate light propagation.[5,6] A plane wave from +z towards -z direction is selected as an incident light source. The model geometry is devised according to the cross-section SEM image displayed in Figure 2c. Periodic boundary conditions are used to model x and y directions, perfectly matched layer (PML) is for the z direction. The mesh element size of 2×2×1 nm^3 is utilized to improve simulation accuracy. Absorption power distribution is monitored by extracting from the 2-D xz- or yz-plane.

Passive radiative heating test: The passive radiative heating test was carried out indoors, in which a DC power supply was connected to a silicon rubber heating plate, providing a constant heat flux of 110 W/m^2 for simulating the human body metabolic heat. A piece of

artificial skin with an emissivity close to human skin was stuck on the heating plate to simulate human skin. Subsequently, the silicone rubber heating plate was wrapped with textiles, and a K-type thermocouple was used to measure the skin surface temperature under the textiles.

Solar heating test on artificial skin: Photo-to-thermal conversion tests were done outdoors at Lanzhou Institute of Chemical Physics, China (103:50 E, 36:03 N). A piece of artificial skin was attached to a rectangular glass slide to simulate human skin, with a thermocouple being fixed on the insulating tape. The surface of the glass slide was covered with textiles, and the edges were sealed with heat-resistant tape. The glass slide was fixed on the melamine foam board for heat insulation. The melamine foam board was adhered with an aluminum foil to reduce heat transfer through conduction and radiation from the ambient. A thermocouple for ambient temperature measurement is attached to the side wall of the foam beneath the skin simulator and sheltered from sunlight. A thermometer and a digital anemometer were utilized to real-time monitor the parameters, including solar irradiance, wind speed, ambient temperature, and relative humidity at the place where the experiment was carried out.

Solar heating test on human body: The demonstration was performed in compliance with a protocol that was approved by the Human Research Ethics Committee at the Lanzhou Institute of Chemical Physics (No. KJZLZD-4). Our warming textile and pristine cotton were attached on a skin of a volunteer. Radiated by natural sunlight, their surface temperature of skin are recorded by a thermocouple. A thermometer and a digital anemometer were utilized to real-time monitor the parameters, including solar irradiance, wind speed, ambient

temperature, and relative humidity at the place where the experiment was carried out. The informed written consent of the participant has been obtained.

Water vapor transmission rate test: The test was performed using ASTM E96 with modification. A beaker filled with 50 ml distilled water was sealed by those textile samples respectively using rubber bands. The sealed beaker was then put into an environmental chamber whose temperature was kept at 25 °C and relative humidity inside at around 46%. The beaker was weighed periodically with an electronic balance. The water vapor transmission rates were calculated from the mass loss, which was equal to the mass of evaporated water.

Mechanical test: A New SANS universal material testing system (CMT4304) was used to evaluate the tensile performance of those textiles, equipped with a 1 KN load cell. The samples with a working area of 1 cm wide and 8 cm long were manufactured for the tensile test; the rate of cross-head motion was fixed at 10 mm/min. All the tests were performed at room temperature.

Wicking test: This test procedure is based on AATCC TM 197 with modification. The textile samples were cut into 2-cm-wide strips and dipped in distilled water. The water climbs up the sample due to capillary force. The climbing distance in the duration of 10 s was measured for the textile samples.

Water contact angle measurement: The static water contact angles were measured by a DropMeter A-200 contact angle system (MAIST Vision Inspection & Measurement Co. Ltd., China) in the ambient environment to evaluate the wettability of the textile

Supplement 2. Heat transfer model analysis

The heat transfer analysis for outdoor radiative warming is based on the one-dimensional steady-state heat transfer model,[7,8] which is carried out to determine the total heat dissipation rate of the human body wearing textile of different optical properties (Figure S28). In this model, sunlight illumination, metabolic heat generation, thermal radiation, conduction, and convection are included to simulate the heat dissipation from the body to ambient air. Among them, the heat gain of the human body is from solar radiation and metabolic heat generation. Heat radiation, conduction, and convection are included to simulate the heat dissipation from a clothed human body to the ambient air. The human body is assumed to be in a sedentary state with a uniform skin temperature and heat generation. All optical properties are assumed to be gray and diffuse. The skin and environment are assumed to be an ideal blackbody emitter and absorber. All radiative view factors are equal to 1. Internal scattering and self-absorption effects are neglected within the cloth. It is assumed that the absorption and emission profile is linear within the cloth. The primary unknown variables in this model are the inner surface textile temperature T_i, the outer surface textile temperature T_o, and the environment temperature T_e.

The energy balance at the skin surface:

$$q_{gen} + q_{rad,i} + \tau_i \cdot \varepsilon_s \cdot q_{amb} + \tau_u \cdot a_s \cdot q_{sun} = q_{conv,s} + a_i \cdot q_{rad,s} \quad \text{(S6)}$$

The energy balance at the textile outer surface:

$$\alpha \cdot q_{sun} + \alpha_i \cdot q_{rad,s} + \alpha_o \cdot q_{amb} + q_{conv,i} = q_{rad,o} + q_{rad,i} + q_{conv,e} \quad \text{(S7)}$$

Where q_{gen} is the metabolic heat generation rate per unit area, $q_{rad,i}$, $q_{rad,s}$, q_{atm}, and $q_{rad,o}$ represent the thermal radiation of textile inner surface, skin, ambient environment, and textile

outer surface, respectively. $q_{conv,s}$ is the convective heat flux from the skin to the air gap, τ_i is the IR transmittance of the textile. ε_s is the emittance of the skin, ε_o is the emittance of the textile outer surface, a_s is the solar absorptance of the skin, τ_u is the transmittance of the textile, q_{sun} is the solar irradiance, $q_{conv,e}$ is the convective heat flux from the textile to the ambient environment, $q_{conv,s}$ is the convective heat flux from the air gap to the textile, r_{i} is the IR reflection of the inner surface of the textile, α is the solar absorption of the outer surface of the textile, r_o is the IR reflection of the outer surface of the textile. The calculations of these parameters are:

$$q_{rad,s} = \varepsilon_s \int I_b(\lambda,\ T)\, d\lambda \qquad \text{(S8)}$$

Thermal radiation of textile can be divided into thermal radiation to the ambient environment (q_{in}) and outer space (q_{out}) through an atmospheric window.

$$q_{rad,o} = q_{out} + q_{in} = \varepsilon_o \int I_b(\lambda,\ T)\tau(\lambda) d\lambda + \varepsilon_o \int I_b(\lambda,\ T)(1-\tau(\lambda)) d\lambda \qquad \text{(S9)}$$

Where $\tau(\lambda)$ is the transmittance of the atmospheric window.

$$q_{rad,i} = \varepsilon_i \int I_b(\lambda,\ T)\, d\lambda \qquad \text{(S10)}$$

$$q_e = \varepsilon_e \int I_b(\lambda,\ T)\, d\lambda \qquad \text{(S11)}$$

$$q_{conv,e} = h_e (T_o - T_e) \qquad \text{(S12)}$$

Due to the moving of textile, the convective heat transfer inside the air gap cannot be ignored. The air temperature inside the air gap T_{gap} is defined as the average temperature of the skin and inner surface of the textile. The convective heat transfer coefficient h_{gap} is determined by the Nusselt number. Therefore, the convective heat transfer inside the air gap can be calculated as:

$$q_{conv,i} = h_{gap} \left(T_{gap} - T_i\right) \qquad \text{(S13)}$$

$$q_{conv,s} = h_{gap}\left(T_s - T_{gap}\right) \quad \text{(S14)}$$

$$T_{gap} = \frac{T_i + T_s}{2} \quad \text{(S15)}$$

To determine the temperature profile within the textile, heat conduction and radiative heat transfer must be included in the heat transfer analysis. If a differential volume element is taken within the cloth, the heat equation will take the following form,

$$\kappa_t \frac{\partial^2 T}{\partial x^2} - \frac{\partial}{\partial x}\left(q_{rad}\right) = 0 \quad \text{(S16)}$$

where κ_t is the textile thermal conductivity and q_{rad} is the net radiative transfer within the textile. The net radiative heat transfer will consist only of incident radiative absorption and outgoing radiative emission as follows,

$$\kappa_t \frac{\partial^2 T}{\partial x^2} = \frac{\partial}{\partial x}\left(q_{rad,i'}\right) + \frac{\partial}{\partial x}\left(q_{rad,o'}\right) + \frac{\partial}{\partial x}\left(q_{rad,s'}\right) + \frac{\partial}{\partial x}\left(q_{amb'}\right) + \frac{\partial}{\partial x}\left(q_{rad,sun'}\right) \quad \text{(S17)}$$

We assume the absorption and emission profile to be linear as follows,

$$q_{rad,i}\left(x\right) = A \cdot x + B \quad \text{(S18)}$$

where A and B are unknown coefficients that will depend on the boundary conditions assumed for each radiative heat flux, and the boundary conditions are as follows,

$$\begin{aligned} &T\left(x=0\right) = T_i \\ &-\kappa_t \frac{\partial T}{\partial x}\left(x=0\right) = q_{conv,i} \end{aligned} \quad \text{(S19)}$$

Therefore, the final temperature relation can be derived,

$$T_o = \frac{t}{2\kappa_t}\left(q_{rad,i} + q_{rad,o} - a_i \cdot q_{rad,s} - a_o \cdot q_{amb} - \alpha \cdot q_{sun}\right) - \frac{2\kappa_a t}{\kappa_t t_a}\left(T_s - T_i\right) + T_i \quad \text{(S20)}$$

Table S1 Input parameters for the heat transfer model analysis.

Symbol	Definition	Value	Unit
q_{gen}	Metabolic heat generation flux	110	$W{\cdot}m^{-2}$
q_{solar}	Solar irradiation power density	0 ~ 800	$W{\cdot}m^{-2}$
T_s	Skin temperature	306	K
k_a	Thermal conductivity of air	0.03	$W{\cdot}m^{-1}{\cdot}K^{-1}$
k_t	Thermal conductivity of textile	0.035	$W{\cdot}m^{-1}{\cdot}K^{-1}$
h_e	Convective heat transfer coefficient between textile and ambient	15	$W{\cdot}m^{-2}{\cdot}K^{-1}$
h_{gap}	Convective heat transfer coefficient between skin and inner surface textile	0.4	$W{\cdot}m^{-2}{\cdot}K^{-1}$
t_a	Air gap Thickness	0.3	mm
t	Textile Thickness	0.233	mm
σ	Stefan-Boltzmann Constant	$5.67*10^{-8}$	$W{\cdot}m^{-2}{\cdot}K^{-4}$
ε_e	Emittance of environment	1	unitless
ε_s	Emittance of skin	1	unitless
ε_i	Emittance of inner surface textile	0.8	unitless
α_i	Absorbance of inner surface textile in infrared region	$\alpha_i=\varepsilon_i=0.8$	unitless

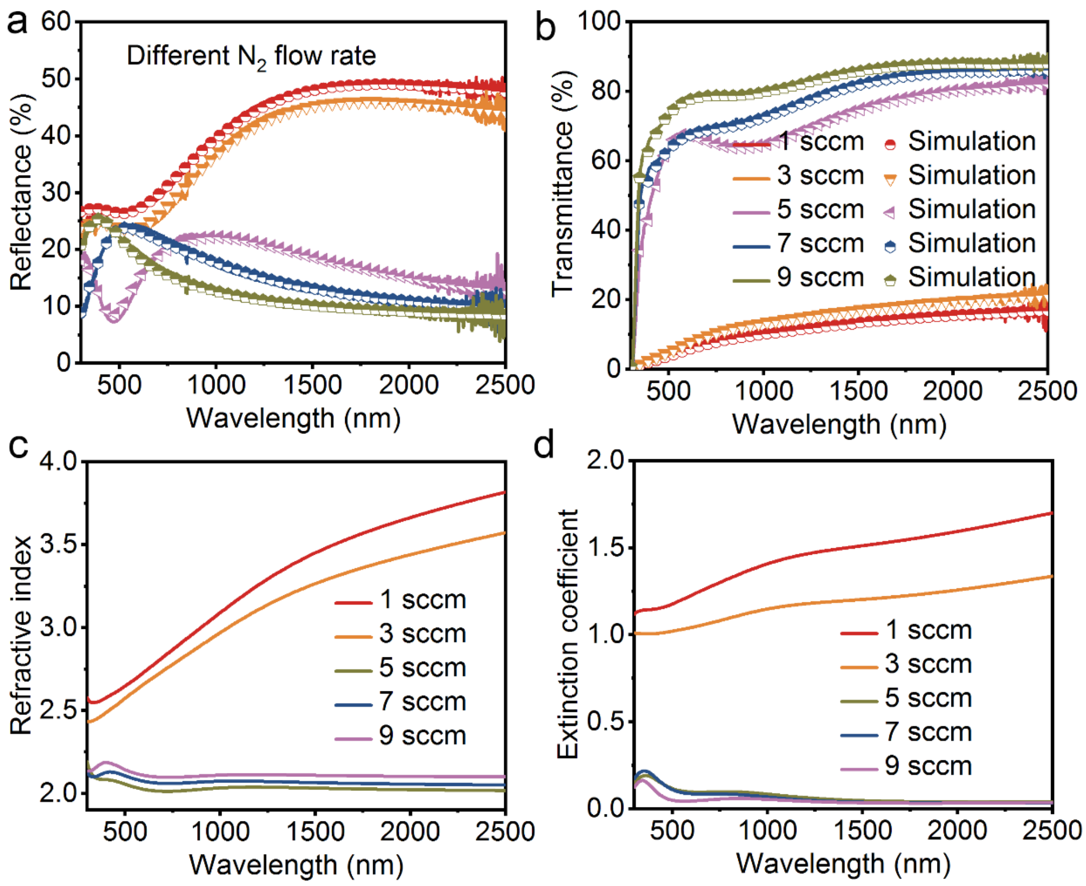

Figure S1. Measured and fitted (a) reflectance and (b) transmittance spectra of ZrNbMo-Al-N films on a glass substrate with different N_2 flow rates. Calculated (c) refractive index and (d) extinction coefficient of corresponding films.

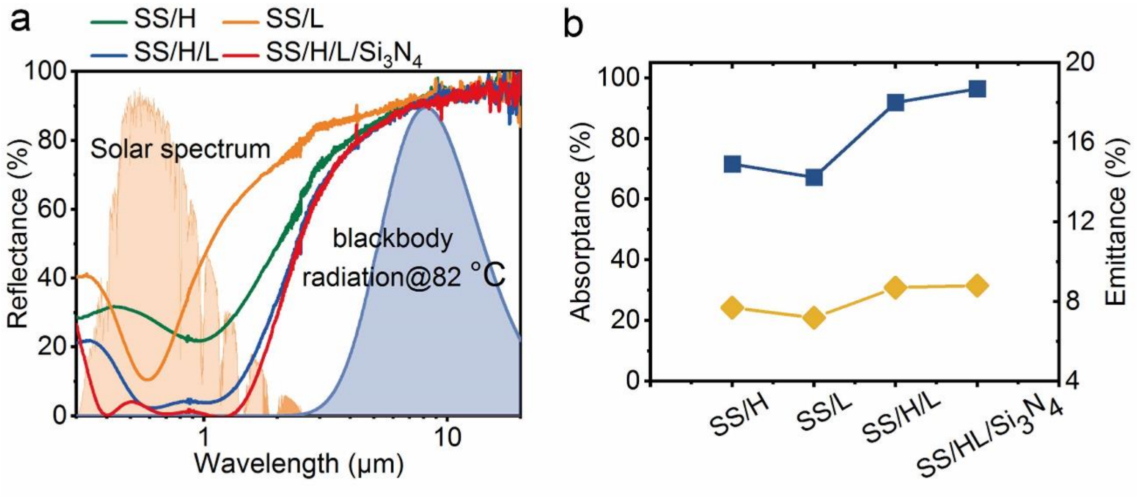

Figure S2. (a) Reflectance spectra of layer-added coating and (b) corresponding solar absorptance and emittance.

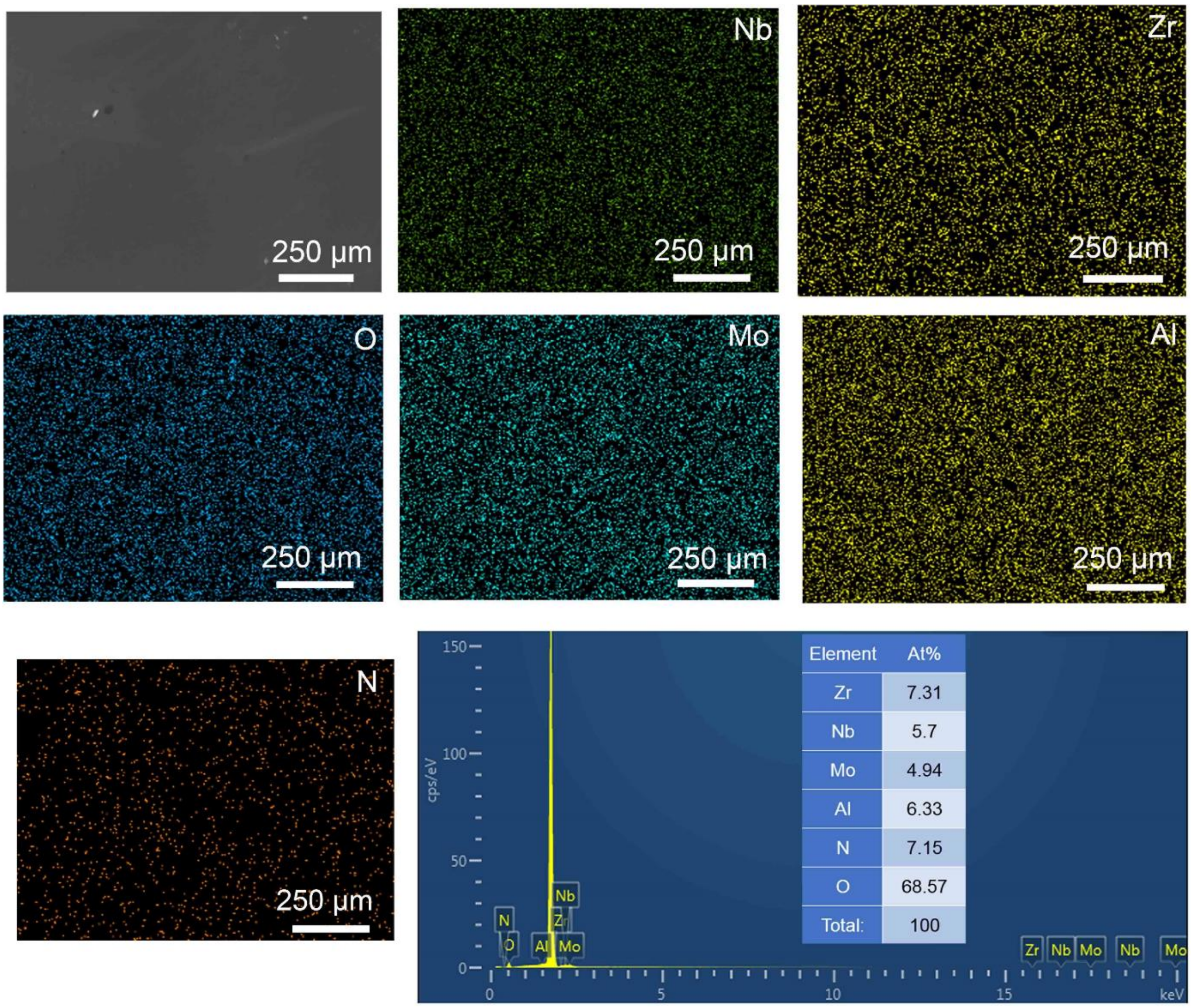

Figure S3. Surface SEM morphology and corresponding region elemental mappings (Nb, Zr, O, Mo Al, and N) and energy dispersive X-ray (EDX) of HMVF layer.

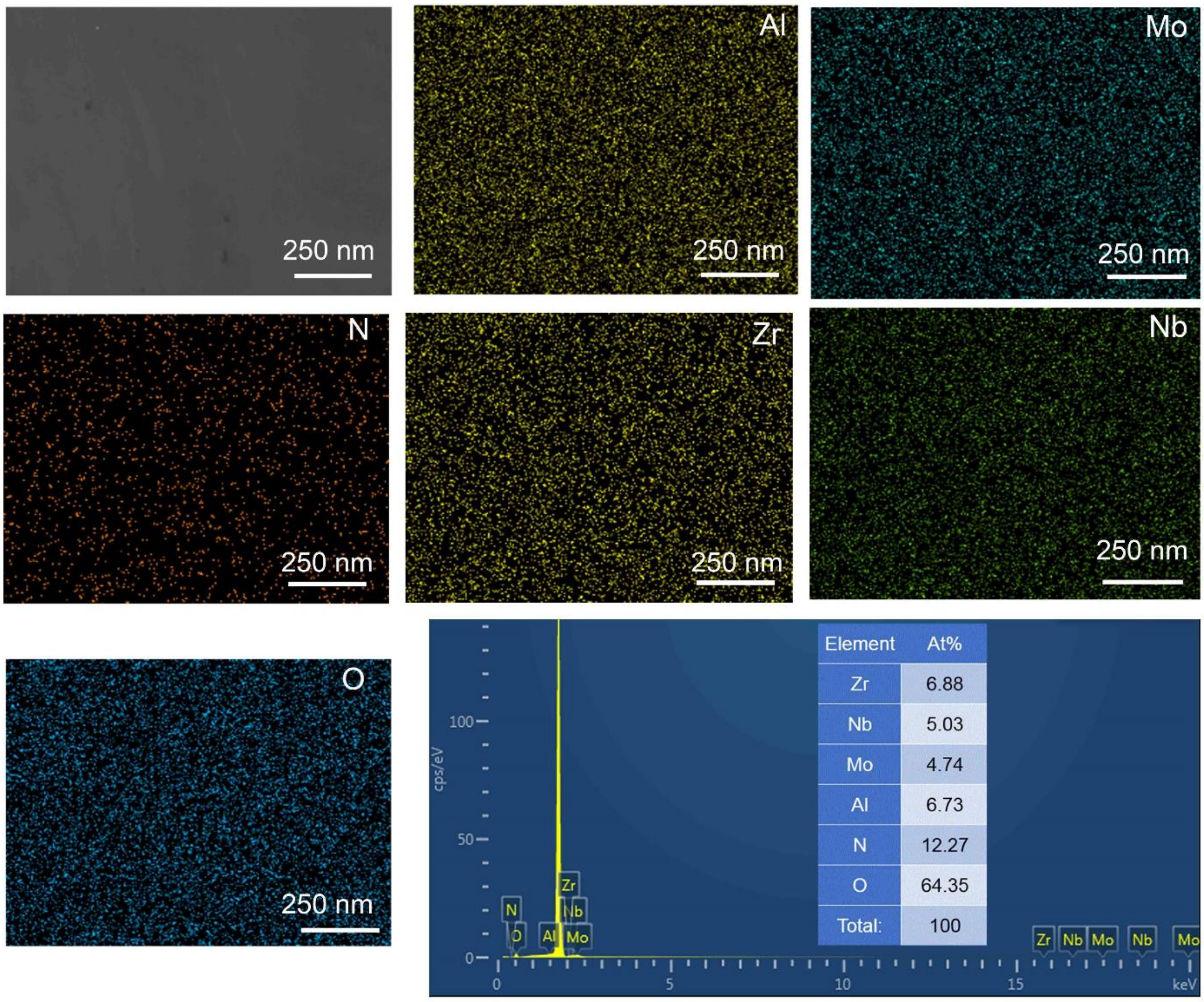

Figure S4. Surface SEM morphology and corresponding region elemental mappings (Nb, Zr, O, Mo Al, and N) and energy dispersive X-ray (EDX) of LMVF layer.

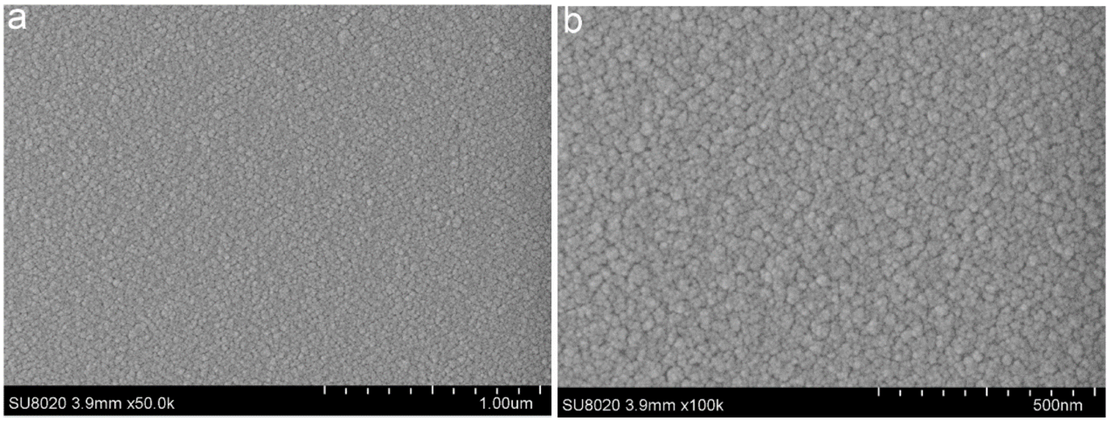

Figure S5 Surface SEM morphologies of as-deposited ZrNbMo-Al-N based absorber on a Si substrate.

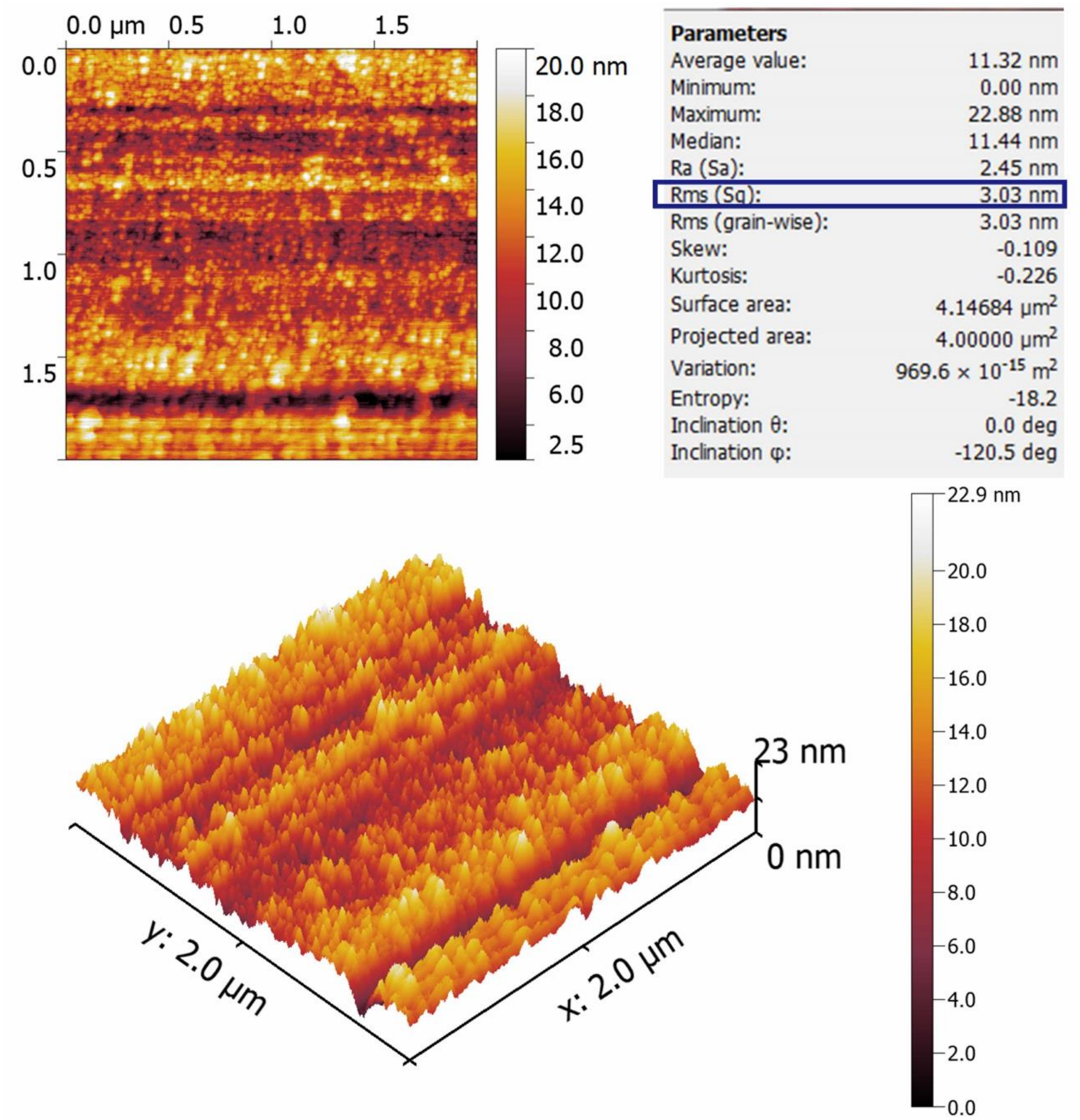

Figure S6. Surface 3-d AFM morphology of as-deposited ZrNbMo-Al-N based absorber.

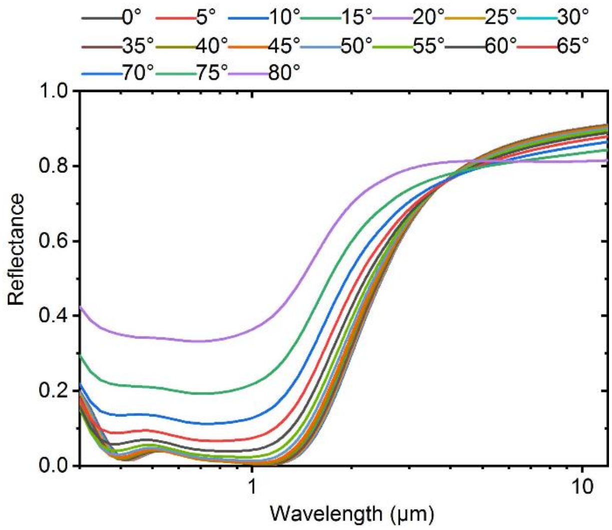

Figure S7. Reflectance spectra of the as-deposited absorber with different incidence angles.

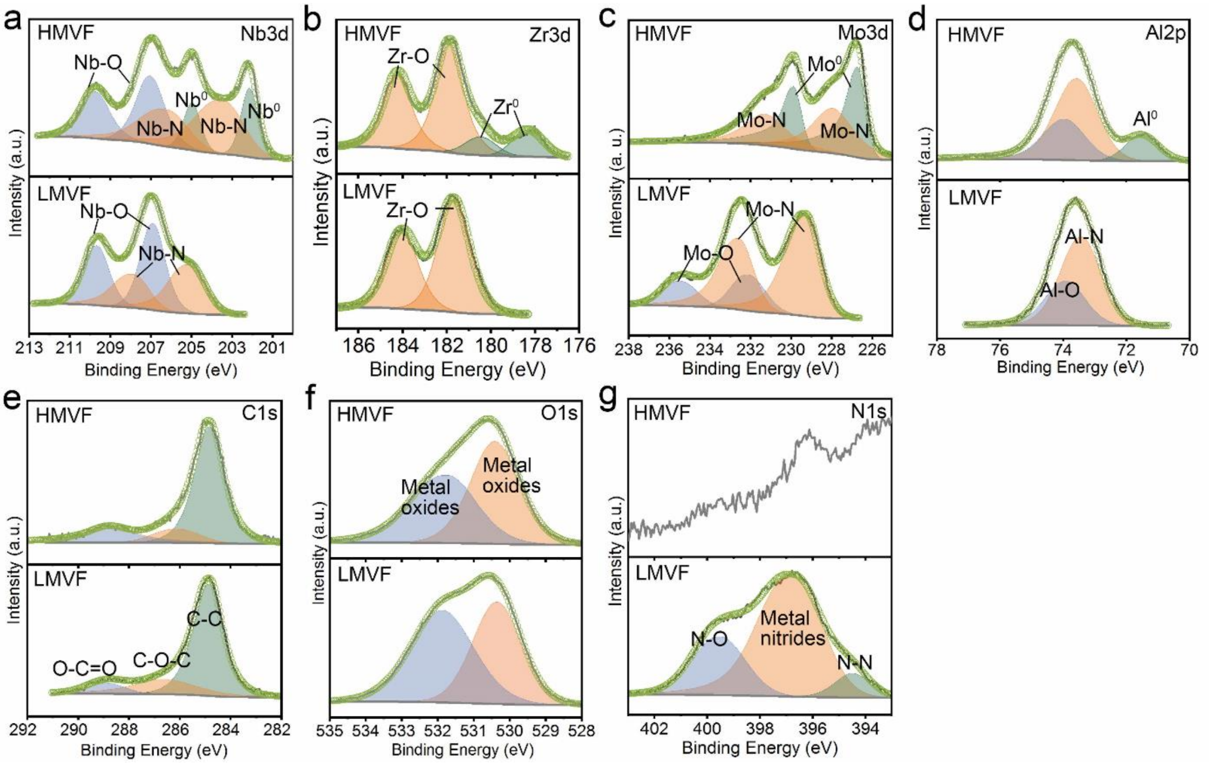

Figure S8 (a) High-resolution XPS spectra of Nb 3d, Zr 3d, Mo 3d, Al 3d, C 1s, O 1s, and N 1s of the as-deposited HMVF and LMVF films.

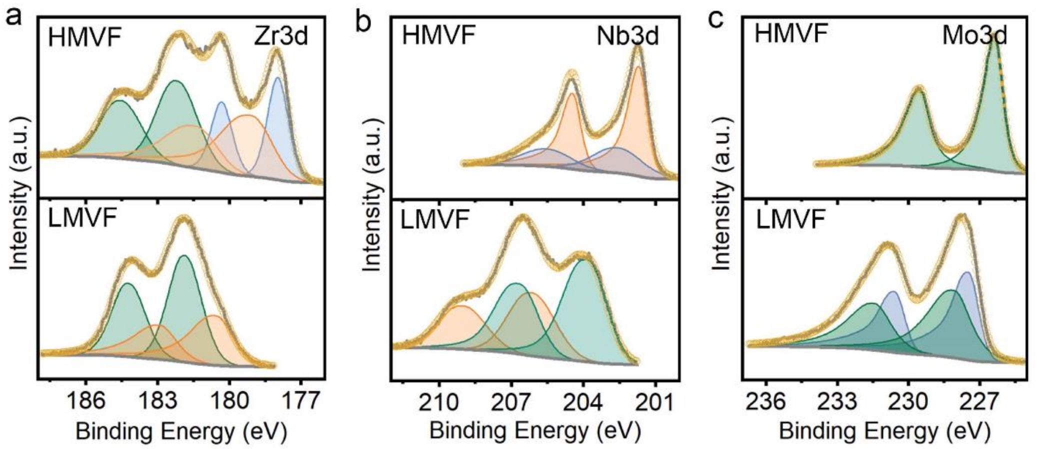

Figure S9 (a) High-resolution XPS spectra of Nb 3d, Zr 3d, and Mo 3d of the HMVF and LMVF films after etching 20 nm.

Figure S9 displays the XPS high-resolution spectra of the etched HMVF and LMVF films. The Zr 3d high-resolution spectrum of HMVF demonstrates the presence of Zr-O (182.2 eV, 184.5 eV), (Zr-N (179.1, 181.4 eV), and Zr^0 (178.0, 180.3 eV) moieties.[9] In comparison, the Zr 3d peaks shift to higher binding energies for LMVF, coordinating as Zr-O (181.9, 184.2 eV) and Zr-N (180.6, 182.9 eV) due to the increased N_2 flow rate. The deconvolution of the Nb 3d peak clearly demonstrates the existence of Nb-N (202.6, 205.5 eV) and Nb^0 (201.8, 204.5 eV) for HMVF;[10] the Nb 3d peaks of LMVF at 206.1 and 209 eV correspond to Nb-O,[11] while the peaks at 203.8, 206.7 eV belong to Nb-N. From the curve-fitting of the Mo 3d spectrum of HMVF, the peaks at 226.4 and 229.5 eV are attributed to Mo^0;[12] for LMVF, the deconvolution reveals the existence of Mo-N (228.5, 230.6 eV) and Mo-O (228.1, 231.4 eV).[13]

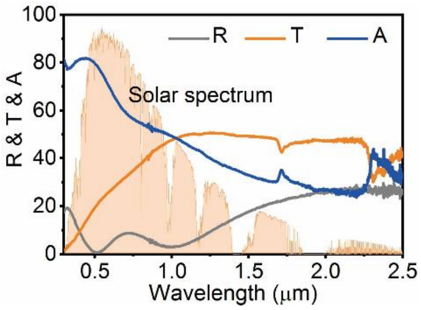

Figure S10. Reflectance, transmittance, and absorptance spectra of ZrNbMo-Al-N based SSF deposited on PET sheet.

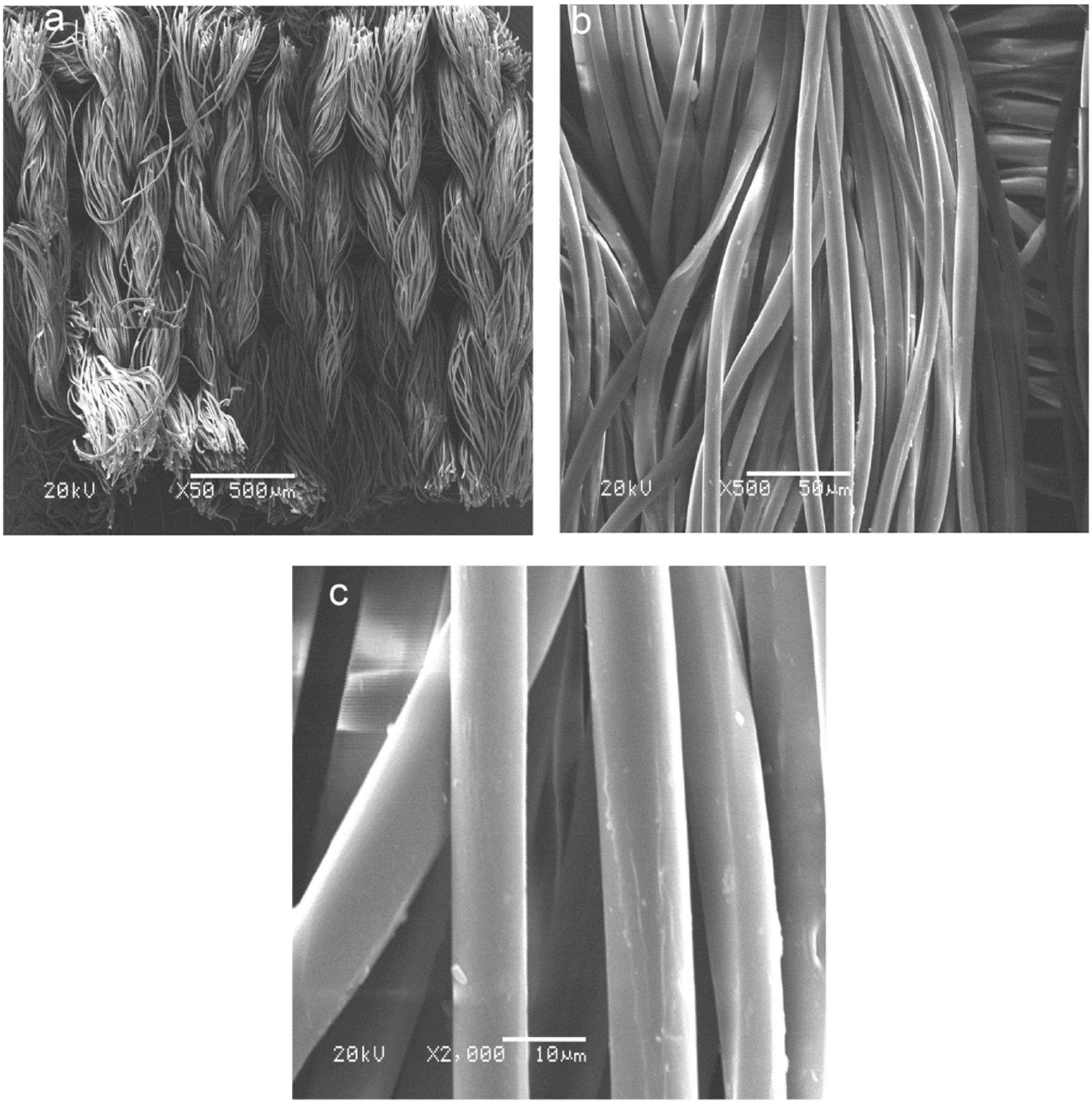

Figure S11. SEM images of pristine cotton with different magnification.

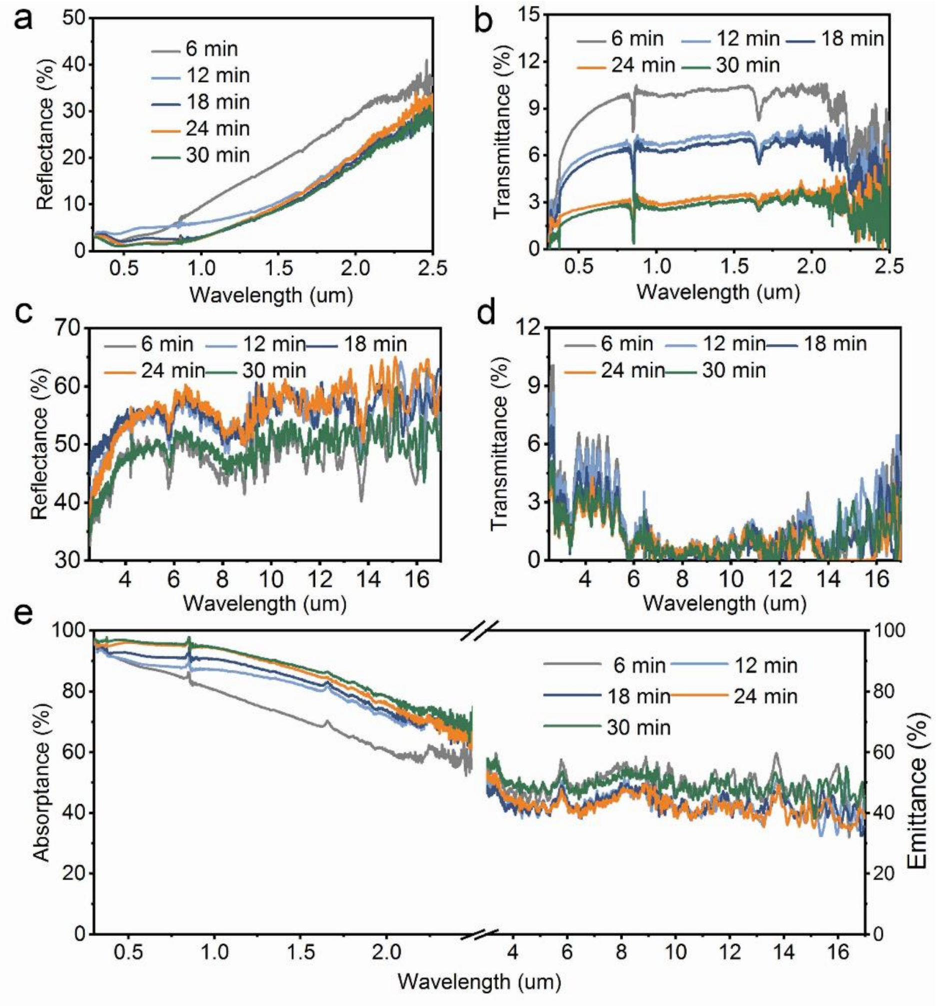

Figure S12. (a) Reflectance and (b) transmittance spectra in the wavelengths of 0.3-2.5 μmof the cotton/Al/SSF with different depositon time, (c) reflectance, and (d) transmittance spectra in the wavelengths of 2.5-17 μm. (e) Solar absorptance and thermal emittance in the wavelengths of 0.3-17 μm.

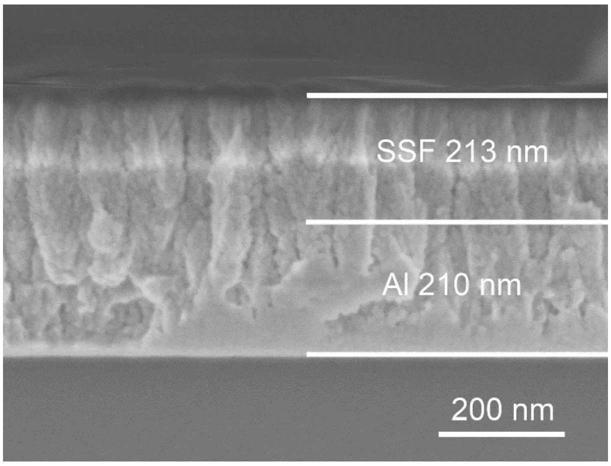

Figure S13. Cross-section SEM morphology of Al/SSF coating on Si substrate.

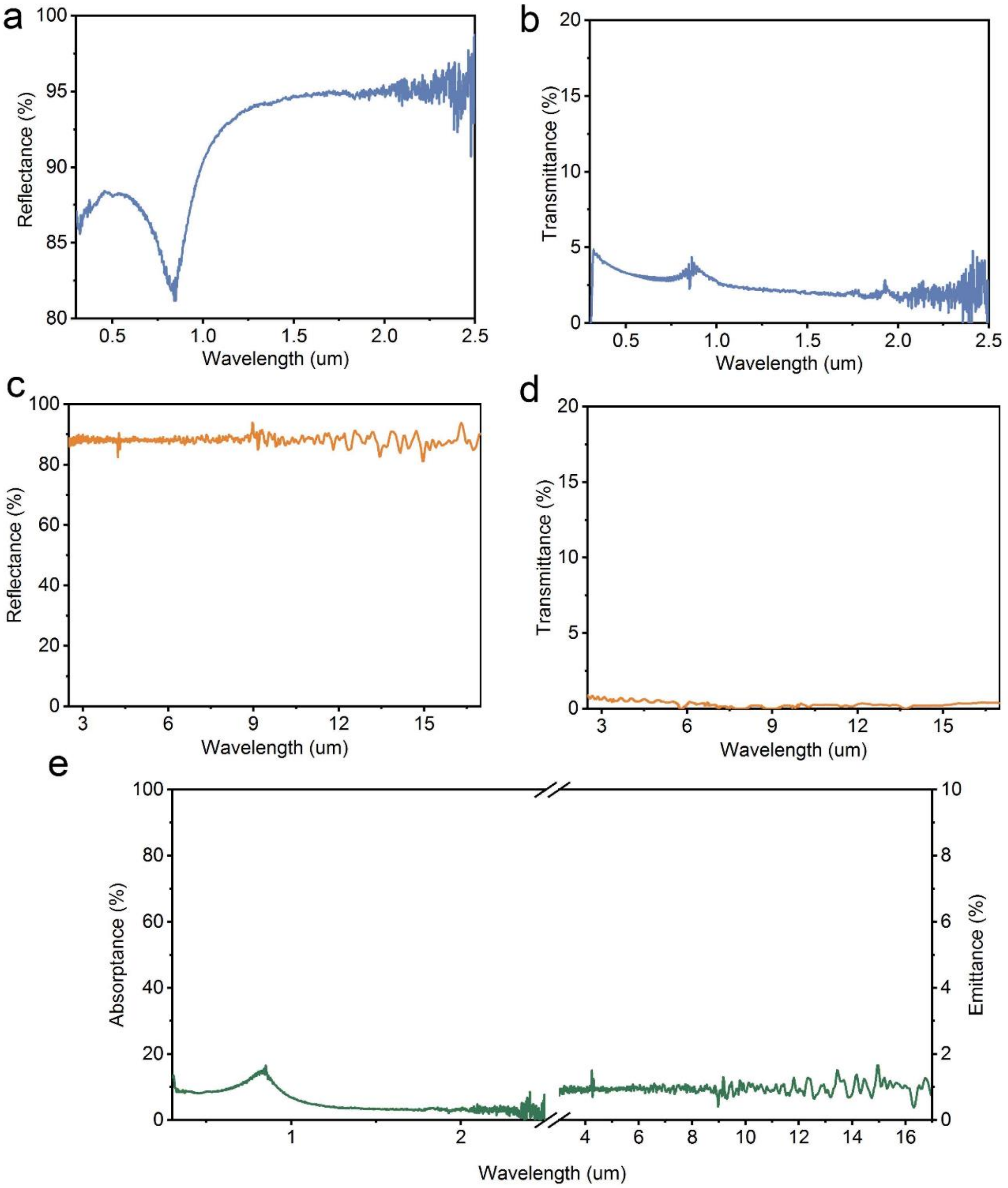

Figure S14. (a) Reflectance and (b) transmittance spectra of the Mylar blanket in the solar spectrum; (c) Reflectance and (d) transmittance spectra of the Mylar blanket in the wavelengths of 0.3-17 μm; (e) Absorptance/emittance spectrum in the wavelength of 0.3-17 μm.

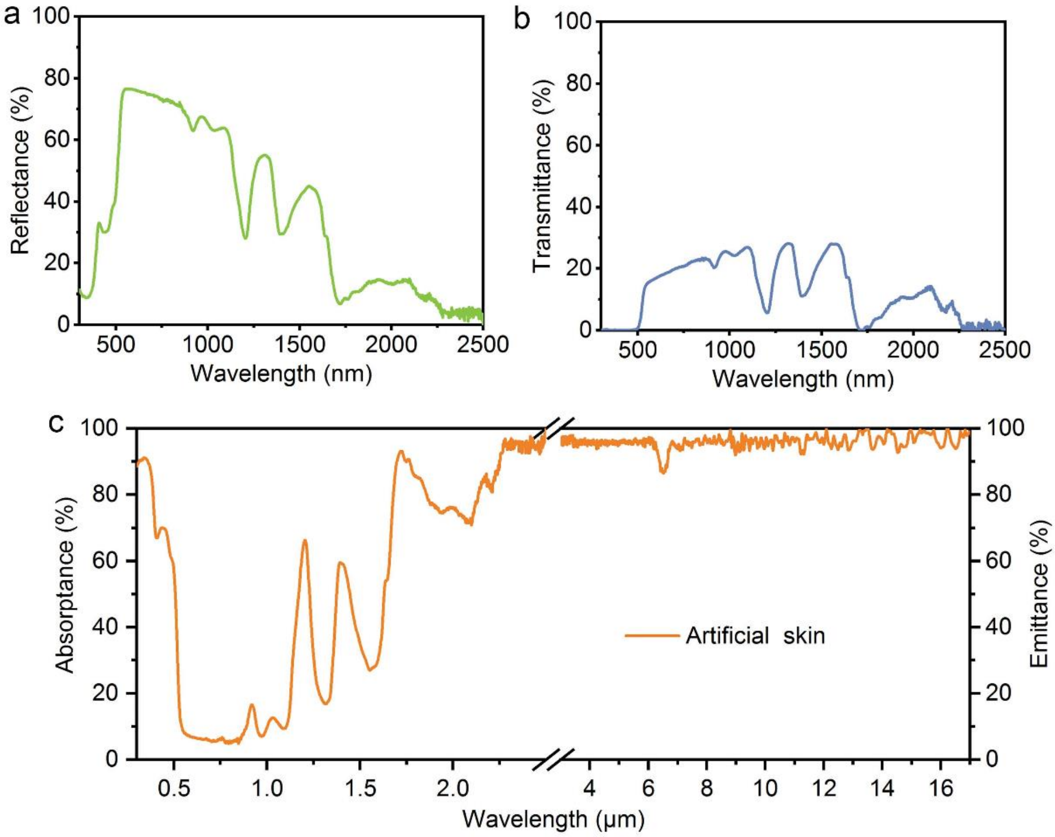

Figure S15. (a) Reflectance and transmittance spectra of the artificial skin in the solar spectrum; (c) Absorptance/emittance spectrum of the artificial skin in the wavelength of 0.3-17 μm.

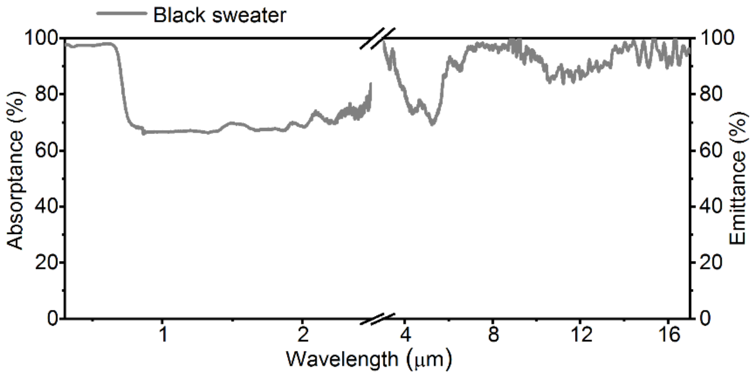

Figure S16. Absorptance/emittance spectrum of the black sweater in the wavelength of 0.3-17 μm.

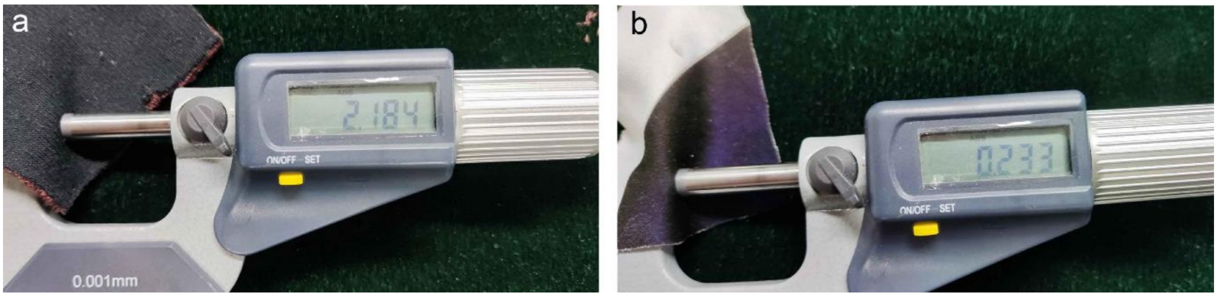

Figure S17. Thickness measurement of (a) black sweater and (b) as-deposited cotton/Al/SSF textile.

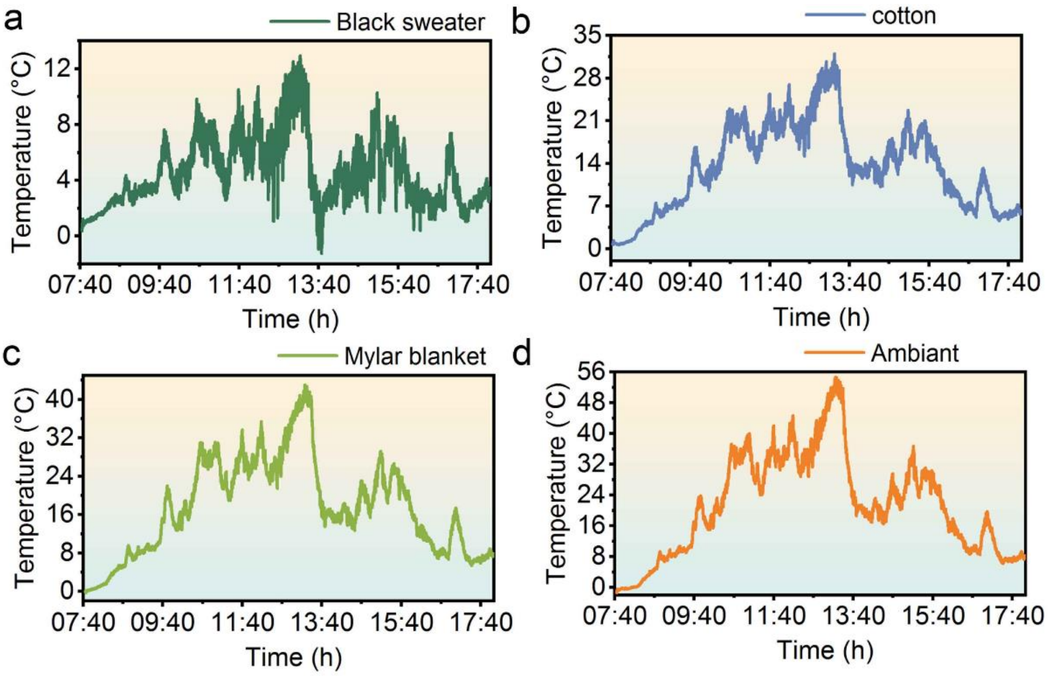

Figure S18. The temperature difference of the cotton/Al/SSF with (a) black sweater, (b) cotton, (c) Mylar blanket, and (d) ambient as covered on artificial skin.

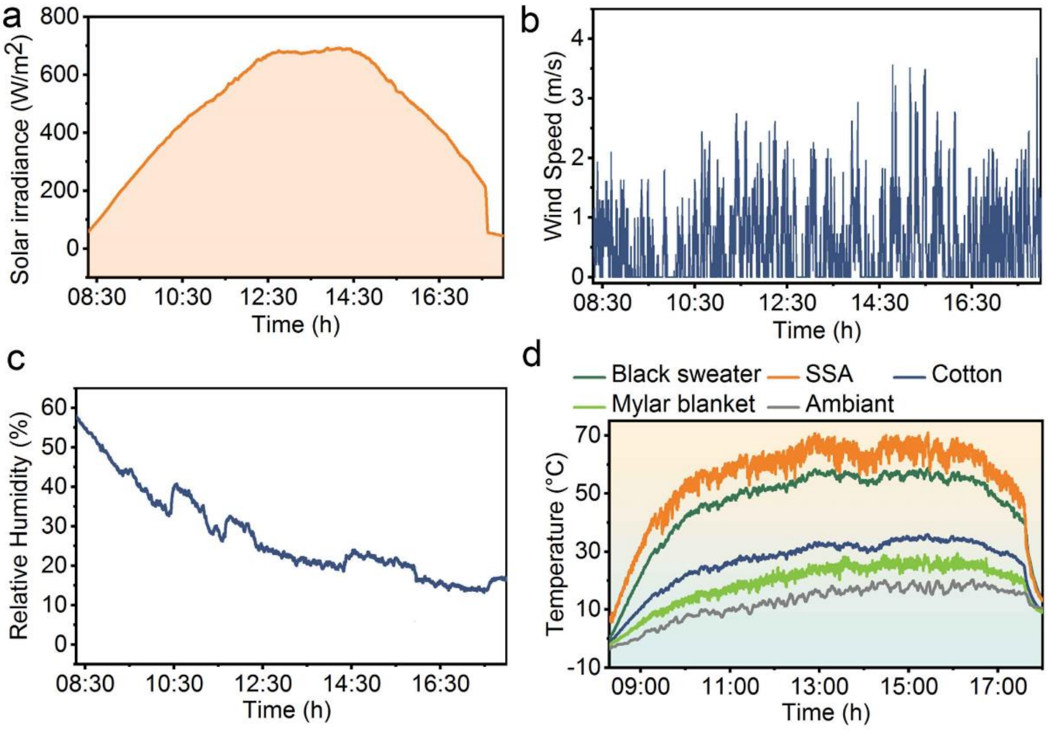

Figure S19. Recorded (a) solar irradiance intensity, wind speed, and relative humidity during the daytime experiment, (d) corresponding real-time temperatures of the skin simulator covered with different textiles.

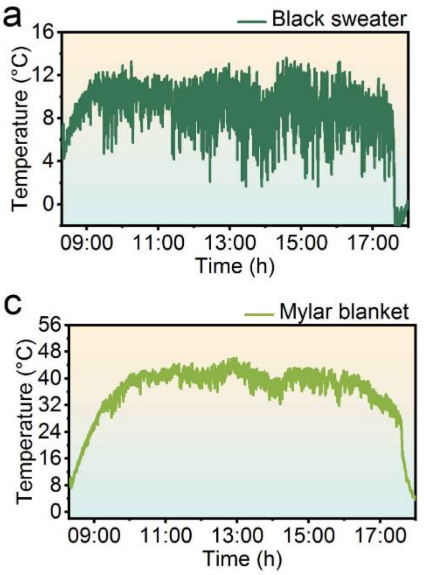

Figure S20. The temperature difference of the cotton/Al/SSF textile with (a) black sweater, (b) cotton, (c) Mylar blanket, and (d) ambient.

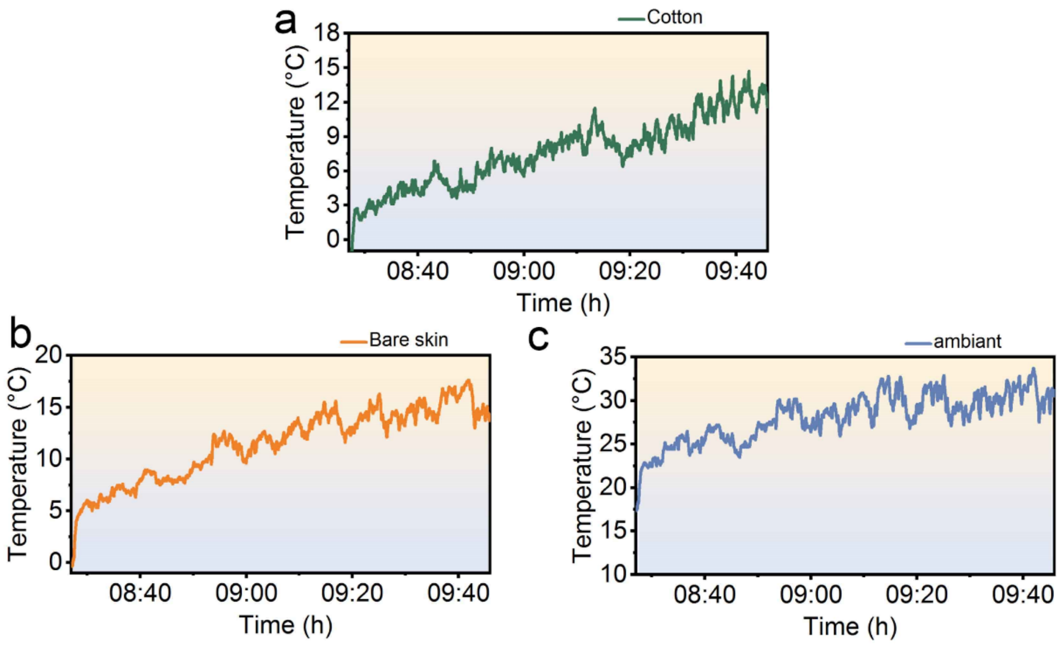

Figure S21. The temperature difference of the cotton/Al/SSF with (a) cotton, (b) bare skin, and (c) ambient as covered on human skin on a sunny day.

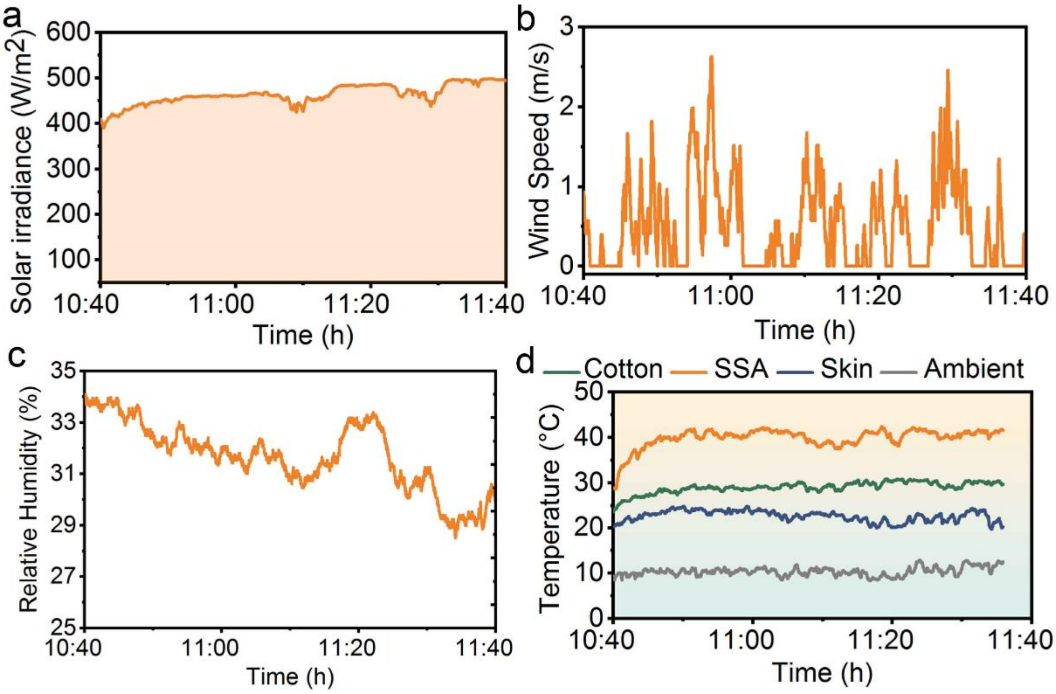

Figure S22. Recorded (a) solar irradiance intensity, (b) wind speed, and (c) relative humidity during the daytime experiment, (d) corresponding real-time temperatures of the human skin covered with different textiles.

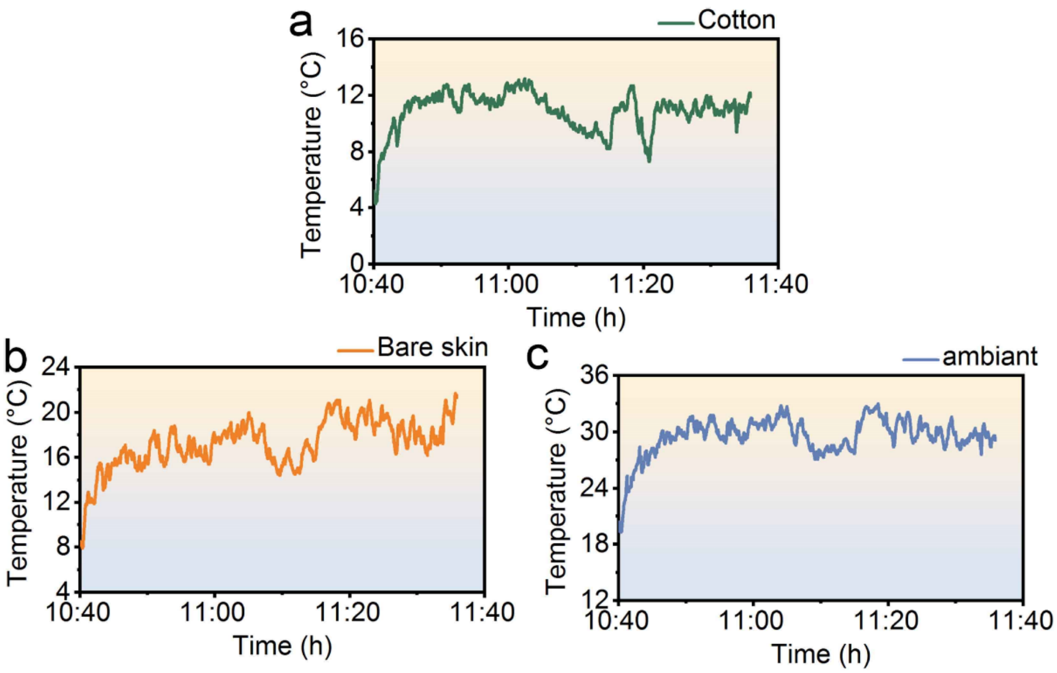

Figure S23. The temperature difference of the cotton/Al/SSF with (a) cotton, (b) bare skin, and (c) ambient as covered on human skin on a cloudy day.

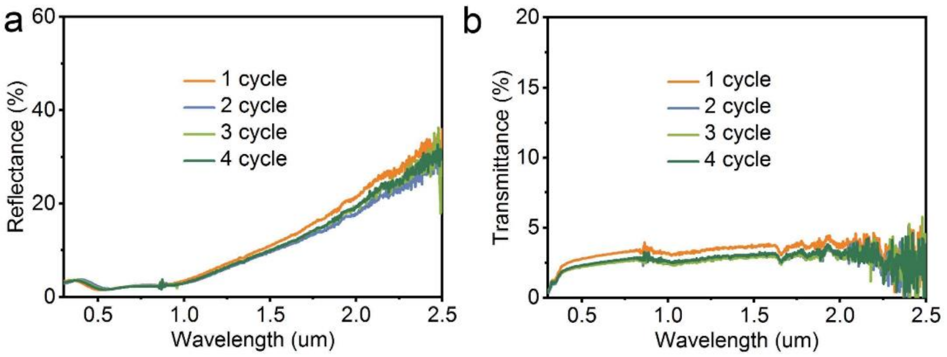

Figure S24. (a) Reflectance and (b) transmittance spectra after different washing cycles.

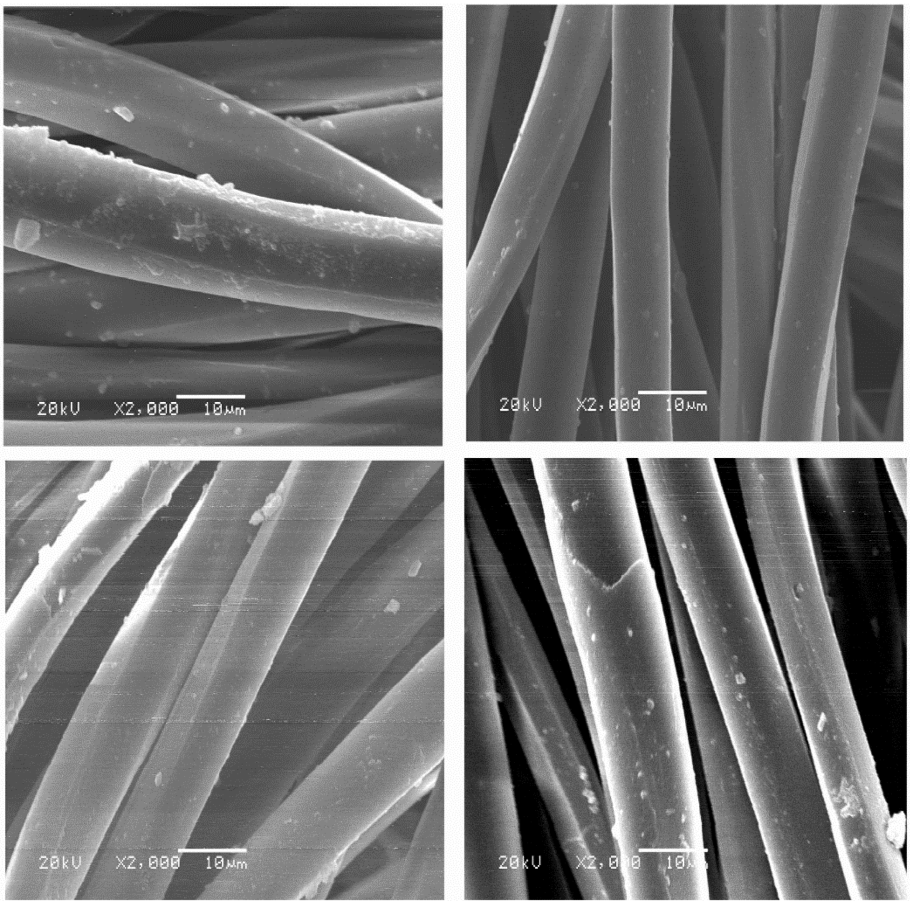

Figure S25. Surface SEM morphology of the cotton/Al/SSF after 1-4 washing cycles.

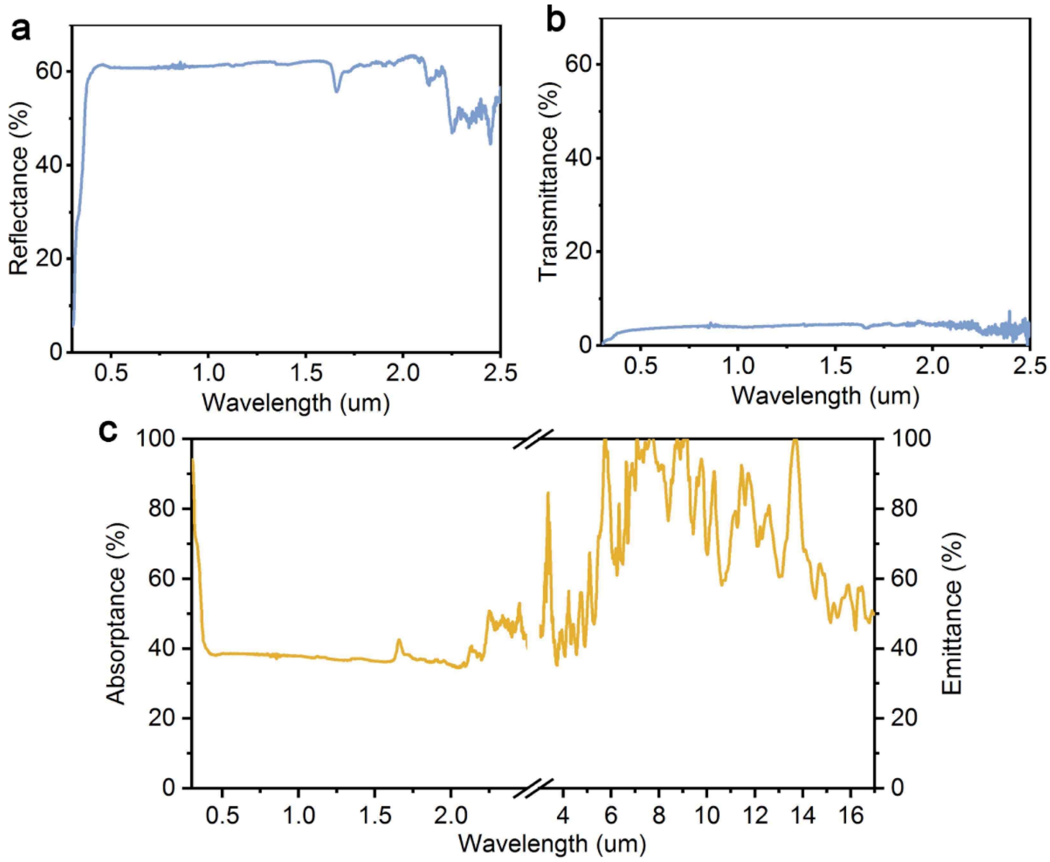

Figure S26. Optical properties of the reverse side for the cotton/Al/SSF.

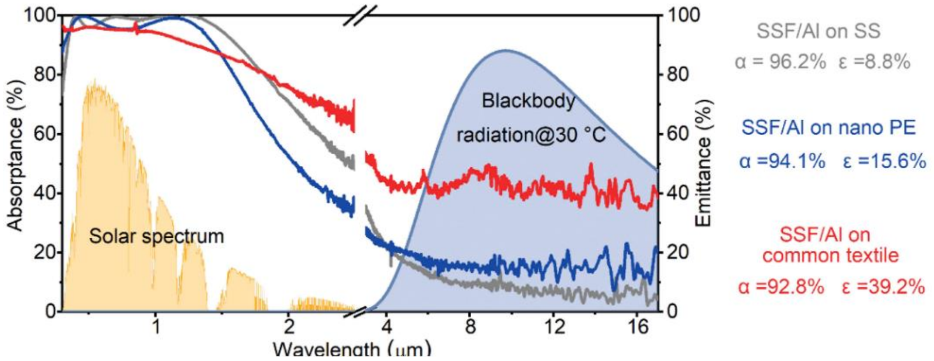

Figure S27. Absorptance spectrum of the optimized SSF on a reflective Al decorated SS, Nano PE, and common textile.

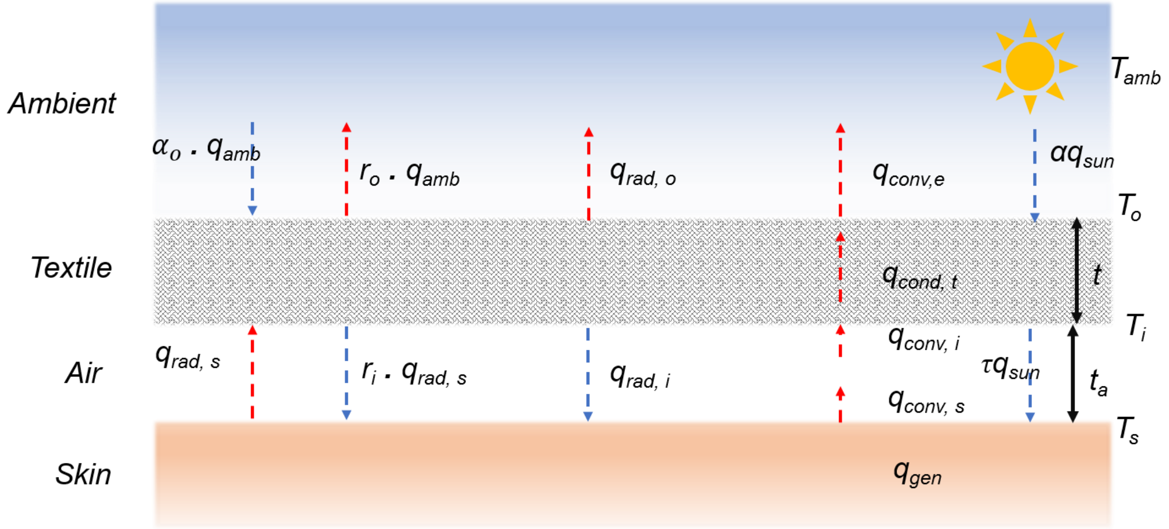

Figure S28. Schematic of one-dimensional heat transfer model.

References

[1] Y. Wu, C. Wang, Y. Sun, Y. Xue, Y. Ning, W. Wang, S. Zhao, E. Tomasella, A. Bousquet, *Solar Energy Materials and Solar Cells* **2015**, *134*, 373.

[2] A. Al-Rjoub, L. Rebouta, P. Costa, N. P. Barradas, E. Alves, P. J. Ferreira, K. Abderrafi, A. Matilainen, K. Pischow, *Solar Energy* **2018**, *172*, 177.

[3] A. Al-Rjoub, L. Rebouta, P. Costa, L. G. Vieira, *Solar Energy Materials and Solar Cells* **2018**, *186*, 300.

[4] Y. Ning, W. Wang, L. Wang, Y. Sun, P. Song, H. Man, Y. Zhang, B. Dai, J. Zhang, C. Wang, Y. Zhang, S. Zhao, E. Tomasella, A. Bousquet, J. Cellier, *Solar Energy Materials and Solar Cells* **2017**, *167*, 178.

[5] X. Wang, H. Hu, X. Li, J. Gao, Z. Wang, L. Liang, H. Zhang, F. Zhuge, H. Cao, *Applied Materials Today* **2020**, *18*, 100533.

[6] J. Wang, Z. Ren, Y. Luo, Z. Wu, Y. Liu, S. Hou, X. Liu, Q. Zhang, F. Cao, *ACS Applied Materials & Interfaces* **2021**, *13*, 40522.

[7] Y. Zhang, Y. Li, K. Li, Y. S. Kwon, T. Tennakoon, C. Wang, K. C. Chan, S.-C. Fu, B. Huang, C. Y. H. Chao, *Nano Energy* **2022**, *95*, 106996.

[8] J. K. Tong, X. Huang, S. V. Boriskina, J. Loomis, Y. Xu, G. Chen, *ACS Photonics* **2015**, *2*, 769.

[9] D. F. Arias, Y. C. Arango, A. Devia, *Applied Surface Science* **2006**, *253*, 1683.

[10] G. Jouve, C. Séverac, S. Cantacuzène, *Thin Solid Films* **1996**, *287*, 146.

[11] A. Darlinski, J. Halbritter, *Surface and Interface Analysis* **1987**, *10*, 223.

[12] M. A. Baker, R. Gilmore, C. Lenardi, W. Gissler, *Applied Surface Science* **1999**, *150*, 255.

[13] A. Wu, Y. Gu, Y. Xie, H. Yan, Y. Jiao, D. Wang, C. Tian, *Journal of Alloys and Compounds* **2021**, *867*, 159066.
